# Composition of the Reconstituted Cell Wall in Protoplast-Derived Cells of *Daucus* Is Affected by Phytosulfokine (PSK)

**DOI:** 10.3390/ijms20215490

**Published:** 2019-11-04

**Authors:** Kamila Godel-Jędrychowska, Katarzyna Maćkowska, Ewa Kurczyńska, Ewa Grzebelus

**Affiliations:** 1 Department of Cell Biology, Faculty of Biology and Environmental Protection, University of Silesia, 28 Jagiellońska Street, 40-032 Katowice, Poland; kamila.godel@gmail.com; 2 Department of Plant Biology and Biotechnology, Faculty of Horticulture, University of Agriculture in Krakow, Al. 29-Listopada 54, 31-425 Kraków, Poland; kmackowska@gmail.com

**Keywords:** AGPs, carrot, cell division, extensin, pectin, plating efficiency

## Abstract

Phytosulfokine-α (PSK), a peptidyl plant growth factor, has been recognized as a promising intercellular signaling molecule involved in cellular proliferation and dedifferentiation. It was shown that PSK stimulated and enhanced cell divisions in protoplast cultures of several species leading to callus and proembryogenic mass formation. Since PSK had been shown to cause an increase in efficiency of somatic embryogenesis, it was reasonable to check the distribution of selected chemical components of the cell walls during the protoplast regeneration process. So far, especially for the carrot, a model species for in vitro cultures, it has not been specified what pectic, arabinogalactan protein (AGP) and extensin epitopes are involved in the reconstruction of the wall in protoplast-derived cells. Even less is known about the correlation between wall regeneration and the presence of PSK during the protoplast culture. Three *Daucus* taxa, including the cultivated carrot, were analyzed during protoplast regeneration. Several antibodies directed against wall components (anti-pectin: LM19, LM20, anti-AGP: JIM4, JIM8, JIM13 and anti-extensin: JIM12) were used. The obtained results indicate a diverse response of the used *Daucus* taxa to PSK in terms of protoplast-derived cell development, and diversity in the chemical composition of the cell walls in the control and the PSK-treated cultures.

## 1. Introduction

The genus *Daucus* belongs to the Apiaceae family recognized as one of the largest families of seed plants [1,2] and includes, following the newest taxonomical revision [2,3], about 40 species. Theoretically, these species may become a valuable source of genetic variability for the cultivated carrot (*D. carota* L. subsp. *sativus* Hoffm)—the only cultivated species of the genus and the most important member of Apiaceae in terms of economic value and nutrition, second to the potato in worldwide vegetable production [2,4]. Since the discovery of somatic embryogenesis in root-derived callus cultures in vitro [5,6], the carrot has become a model species for plant tissue culture systems. Among various plant in vitro techniques, protoplast cultures offer a unique approach useful in crop improvement i.e., protoplast fusion-based somatic hybridization/cybridization (reviewed in Eeckhaut et al. [7] and Wang et al. [8]). Research on *Daucus* protoplast cultures began when parallel successful protoplast isolation from carrot roots [9] and from cell suspensions [10] were reported. Despite the availability of many *Daucus* genetic resources mentioned above, so far only protoplasts isolated from one close relative, i.e., *D. carota* subsp. *capillifolius* (Gilli) Arbizu [11], have been fused in a symmetric/asymmetric way with protoplasts of the cultivated carrot [12,13]. In general, the ability to regenerate plants from fused protoplasts is a fundamental reason for the limited use of somatic hybridization/cybridization. Therefore, before applying this procedure to plant breeding programs, an efficient protoplast-to-plant-system for both fusion partners should be accessible. There are several reports on successful plant regeneration from carrot protoplasts with respect to different types of source tissue [10,14,15] and to the best of our knowledge, only one with regard to wild *Daucus* species, showing extremely different reactions of the examined accessions to the culture, ranging from highly responsive to recalcitrant [16].

Several parameters influence the ability of protoplasts and protoplast-derived cells to express their totipotency and to develop into fertile plants [17]. Re-synthesis of the cell wall is one of the most important key steps in the protoplast development preceding mitotic divisions and initiating the establishment of a protoplast-to-plant system. Protoplast-based approaches are not only a convenient tool for plant improvement but they also provide a good model for studies in plant physiology, pathology, molecular biology, cytogenetic [18] as well as for studying the correlation between cell-wall composition and cell differentiation [19]. It is postulated that the cell-wall composition and the changes to which it is subjected during differentiation, redifferentiation and dedifferentiation reflect morphogenetic events during plant development [19,20,21].

Application of protoplast cultures to plant improvement requires knowledge from different disciplines and familiarity with factors involved in developmental processes. It is known, that among the factors involved in these processes, the cell wall chemical components and their molecular organization are of special interest [19,22]. Different methods have been used to determine the chemical composition during cell wall regeneration [23,24,25]. However, to answer the question of the spatial location of individual chemical components in the wall during the regeneration process, it is necessary to use immunohistochemical methods. Thus, application of monoclonal antibodies (mAbs) against pectic, arabinogalactan proteins (AGPs) and extensins epitopes allows us to indicate the precise distribution of these components in muro [19,21,26,27]. So far our knowledge concerning spatio-temporal distribution of wall components during the wall regeneration is scarce. Among the few existing reports referring to this issue, the presence of pectic, xyloglucan and carbohydrate AGP epitopes in leaf-derived protoplasts of the sugar beet (*Beta vulgaris* L.) was described [19,28]. In later studies, Wiśniewska and Majewska-Sawka [29] confirmed the presence of some AGPs and pectins in the reconstituted cell wall of leaf-derived protoplasts also in *Nicotiana tabacum* L.

Recently, phytosulfokine, a peptidyl plant growth factor, has been recognized as a promising intercellular signaling molecule involved in cellular proliferation and dedifferentiation. It was shown that PSK stimulated and enhanced cell divisions in protoplast cultures of several species (*Beta vulgaris*, *Daucus carota*, *Daucus montevidensis*, *Daucus pussilus* and *Oryza sativa*) leading to callus and proembryonic mass (PEM) formation [16,30,31]. However, the mechanism for such stimulation is not known. As it has been reported that somatic embryogenesis is accompanied by such changes, they might result from the effect on the chemical composition of the walls [21,26,27].

So far, a detailed description of the cell wall components deposition during wall re-synthesis in *Daucus* protoplast cultures and the possible influence of the PSK on that process have not been provided. Thus, the aim of the present study was to investigate the composition of the reconstituted cell wall and to evaluate the effect of PSK on this process in three *Daucus* taxa, with a different taxonomical background i.e., close (subspecies of *D. carota* subsp. *sativus*) and far (wild *Daucus* species) relative of carrot as well as with a different response to protoplast culture conditions. 

## 2. Results

### 2.1. Protoplast Development

**Figure 1:**
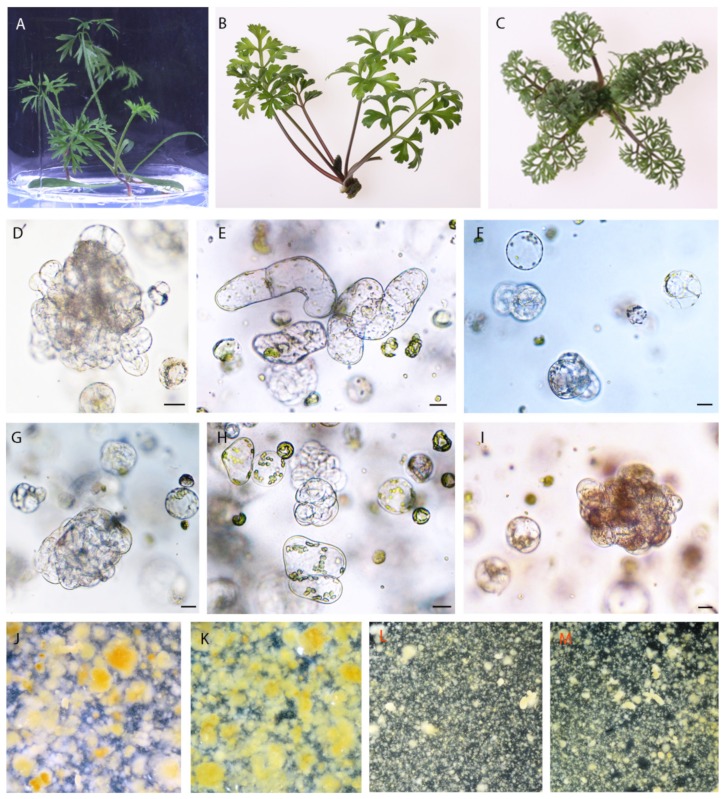
Protoplast cultures of three *Daucus* taxa under control condition and phytosulfokine (PSK) treatment. **A**–**C** donor plants for protoplast isolation of cv. Dolanka, *D. carota* subsp. *gadecaei* and *D. montevidensis*, respectively; protoplast-derived cell colonies after mitotic divisions in 10 day-old control (**D**–**F**) and PSK-treated (**G**–**I**) cultures of cv. Dolanka, *D. carota* subsp. *gadecaei* and *D. montevidensis*, respectively; macrocolonies and somatic embryos in 2 month-old control (**J, L**) and PSK-treated (**K,M**) cultures of cv. Dolanka (**J**–**K**) and *D. carota* subsp. *gadecaei* (**L**–**M**), respectively; bars 20 µm.

The leaves of all studied accessions excised from 2-week-old in vitro grown plants (Figure 1A–C) were an excellent source tissue for protoplast isolation. The applied protoplast isolation procedure combining mechanical and enzymatic treatment released a very large number of protoplasts in the edible carrot (cv. Dolanka) and *D. carota* subsp. *gadecaei* (more than 5 × 10^6^ protoplasts per gram of fresh weight in both accessions, Table 1) and about five fold fewer cells (*p* ≤ 0.05), but still enough even for protoplast fusion, from the wild species *D. montevidensis*. Purified protoplasts of *D. carota* subspecies showed quite a high level of viability reaching about 68%–69% while *D. montevidensis* protoplasts were much more sensitive to applied procedure and exhibited about 20% lower viability (Table 1).

**Figure 2:**
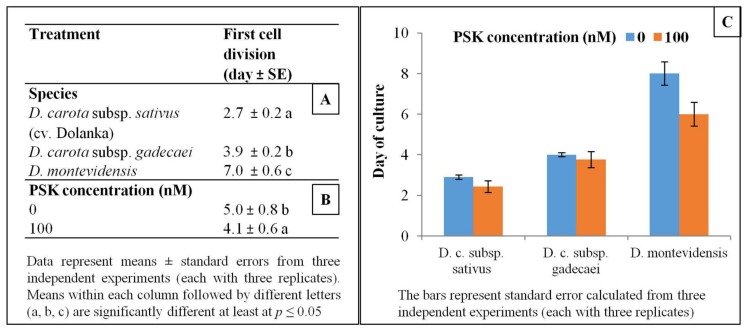
The time point of the first cell division in the control and PSK-treated protoplast cultures of three *Daucus* species—effect of species (**A**), PSK supplementation (**B**) and species and PSK supplementation (**C**) following ANOVA analysis. Means within each column followed by different letters (a, b, c) are significantly different at least at *p* ≤ 0.05

**Figure 3:**
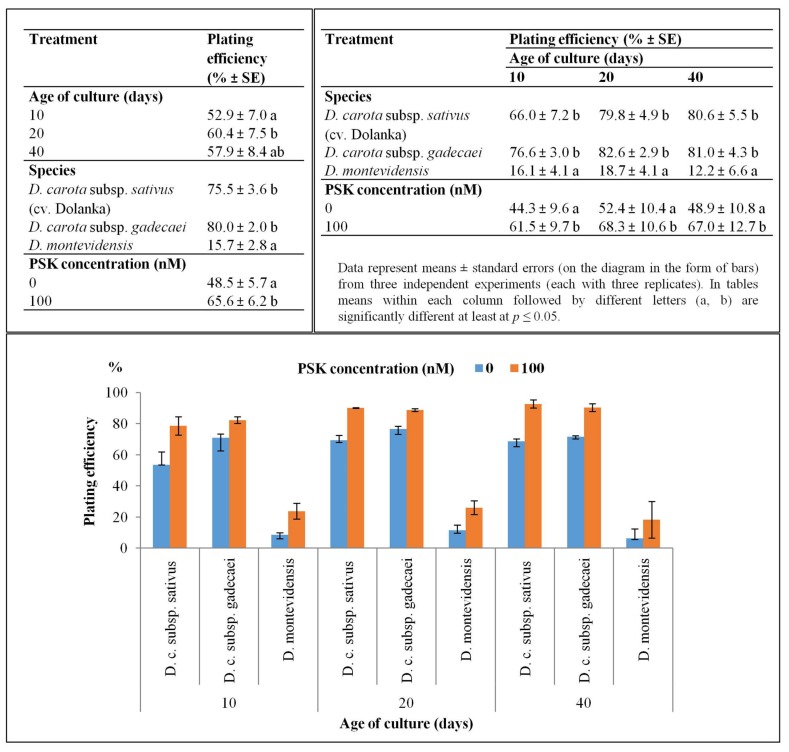
Plating efficiency in control and PSK-treated protoplast cultures of three *Daucus* species. In tables means within each column followed by different letters (a, b) are significantly different at least at *p* ≤ 0.05.

After one day of culture two types of morphological changes in cells of all studied species were observed in transmitted light i.e., cells enlarged in size, and at the same time the shape of the cells gradually altered from the ideal round to oval-like. These symptoms preceded the first cell division, which took place at the earliest in 3-day-old protoplast cultures of Dolanka and at the very latest in 7-day-old protoplast cultures of *D. montevidensis* (*p* ≤ 0.05, Figure 2). In PSK-supplemented cultures the first mitotic division occurred on average one day earlier than in the control cultures. Such a trend exhibiting faster induction of cell divisions in the presence of PSK was recorded for all studied species (Figure 2). In the next days of the culture, systematic mitotic divisions of protoplast-derived cells were most often observed leading to multi-cell aggregates formation (Figure 1D–I and Figure 3). A statistically significant increase in the number of cell aggregates, on average from 53% to 60%, was recorded between the 10th and 20th day of culture, respectively (Figure 3). However, in 40-day old cultures of *D. montevidensis*, both in the control and PSK-treated variants, a slightly decreased number of cell aggregates were scored in comparison to 20-day old cultures (Figure 3). It was the result of degeneration processes manifested by cell and aggregate browning, which finally led to a complete arrest of mitotic activity of the cells in the protoplast cultures of *D. montevidensis*. In general, the protoplast cultures of cv. Dolanka and *D. carota* subsp. *gadecaei* were highly responsive and produced plenty of cell colonies (76–80%) while in the protoplast cultures of *D. montevidensis* the plating efficiency was almost five-fold lower (Figure 3). On average, about 17% more cell colonies were formed in the PSK-treated cultures in comparison to the control variants. In relation to this, after 2 months of culture, only in the case of *D. carota* subspecies were colonies bigger than 500 µm, defined as macrocolonies, and proembryonic mass mostly with globular somatic embryos developed (Figure 1J–M).

### 2.2. Reconstitution of the Cell Wall in the Early Stages of the Protoplast-Derived Cell Cultures

#### 2.2.1. *Daucus Carota* Subsp. *sativus*

**Figure 4:**
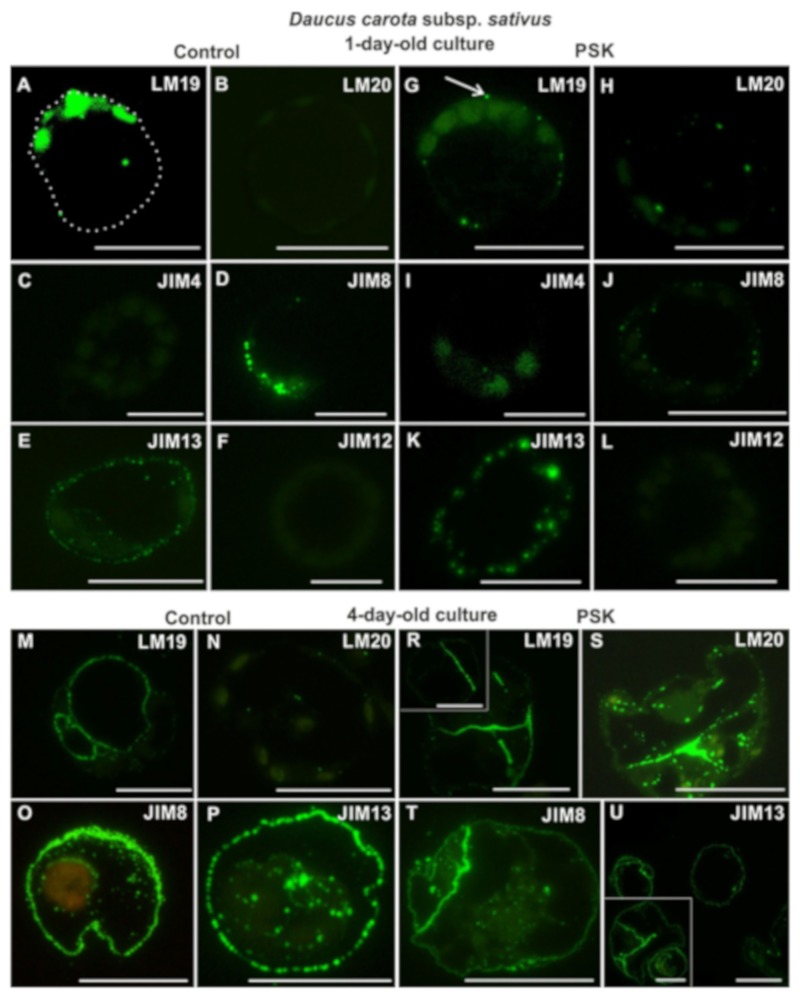
The presence of pectic (LM19, LM20; **A,B,G,H,M,N,R,S**), AGPs (JIM4, JIM8, JIM13; **C,D,E,I,J,K,O,T**) and extensin (JIM12; **F,L**) epitopes during cell wall regeneration in one- and four-day-old protoplast cultures of *Daucus carota* subsp. *sativus* (the white dotted line outlined the border of the protoplast derived cell; the white arrow points to the signal in the regenerating cell wall; the inset in R shows the presence of the pectic epitope only in the walls after the division of the protoplast-derived cells; the inset in U shows the presence of the arabinogalactan protein (AGP) epitope in the new dividing walls; the scale bars = 10 µm).

After one day of culture in control a variant occurrence of the LM19 epitope in the cytoplasm-surrounded plastids of individual cells was detected (Figure 4A). It is interesting that the location of the signals seems to be polarized to one pole of the protoplast-derived cells, which was repetitive in 89%. In the protoplast cultured with addition of PSK, this epitope occurred in the form of single dots evenly distributed in the regenerating wall (Figure 4G). In the case of LM20 epitope, there was no signal in the control (Figure 4B), whereas in the PSK-treated culture it was present in a small amount in the form of dots (Figure 4H). Within the analyzed AGPs epitopes JIM4 was not present in either the control or the PSK-treated cultures (Figure 4C,I). The JIM8 epitope in the control culture was more abundant than in the PSK-treated variant (Figure 4D,J). In the protoplast-derived cells from both types of cultures this epitope was located in cytoplasmic compartments, which means that JIM8 epitope participates in cell wall regeneration from the early stages. It is worth noticing that in the PSK-treated cultures it was clearly located in the tonoplasts of the cells (Figure 4J). The JIM13 epitope occurred abundantly in both the control and the PSK-treated cells, where it was present in greater quantity (Figure 4E,K). The extensin recognized by the JIM12 antibody was not present either in the control or in the PSK-treated cultures (Figure 4F,L).

In 4-day old control cultures the LM19 epitope was abundant in the regenerating cell walls of both the parent protoplast-derived cells and in the new cells formed after divisions (Figure 4M). In the PSK-treated cultures this epitope was also abundant, but only in the division walls (Figure 4R). The LM20 epitope began to be visible in the control culture, in the form of single dotted signals in the regenerating wall as well as in cellular compartments (Figure 4N). In the PSK-treated culture this epitope was abundant both in the new walls and in cellular compartments, but almost absent from the parental protoplasts regenerating the wall (Figure 4S). Both, the JIM8 and JIM13 epitopes were abundant in the walls and in the cellular compartments of the control and PSK-treated culture (Figure 4O–P,T–U). In the PSK-supplemented cultures the epitope recognized by the JIM8 antibody was abundantly present in the walls resulting from divisions of the parent protoplast-derived cells (Figure 4T). The secretion of the epitope detected by JIM8 antibody outside of the cell wall was observed, particularly abundant in the control variant (Figure 4O). There was still no AGP epitope detected by the JIM4 antibody and the extensin epitope in either the control or the PSK-treated cultures.

#### 2.2.2. *Daucus Carota* Subsp. *gadecaei*

**Figure 5:**
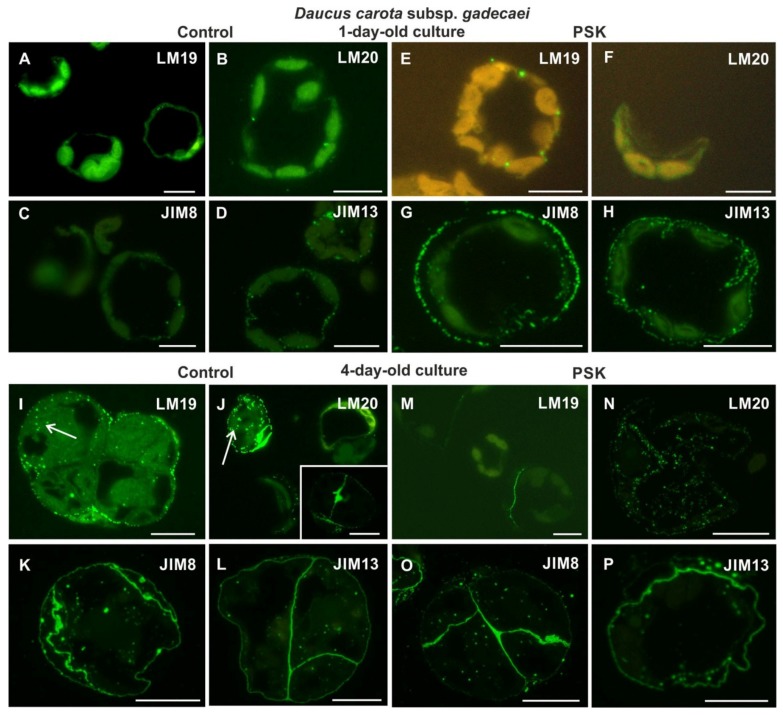
The presence of pectic (LM19, LM20; **A,B,E,F,I,J,M,N**) and AGPs (JIM8, JIM13; **C,D,G,H,K,L,O,P**) epitopes during cell wall regeneration in one- and four-day-old protoplast cultures of *Daucus carota* subsp. *gadecaei* (white arrow on I and J points to epitope presence in cytoplasmic compartments; inset in J—the epitope presence in the new walls after division of protoplast-derived cell; scale bars = 10 µm).

After the first day of culture, the pectic epitope recognized by the LM19 antibody was present in a very small amount, in the form of small dots, both in the walls of the control and the PSK-treated cells (Figure 5A,E). The pectic epitope recognized by the LM20 antibody was detected in the control culture, even in a lesser amount in comparison to the LM19 epitope (Figure 5B), and in the PSK-treated cells it was not detected (Figure 5F). Among the AGP epitopes, only those recognized by the JIM8 and JIM13 antibody were detected, with much higher amount in the cells cultured in the presence of PSK (Figure 5C,D,G,H). Extensin was not detected.

After four days of the culture in the control, the pectic epitope recognized by the LM19 antibody occurred in the outer walls of cell complexes in the form of numerous dots (Figure 5I). Moreover, this epitope was also detected in the cytoplasmic compartments. In the case of the pectic epitope recognized by the LM20 antibody in the cells in which it occurred (because the culture was asynchronous, the cells represented different stages of wall regeneration, some of which did not have this epitope) was located not only in the wall but also in the cytoplasmic compartments (Figure 5J). The epitope appeared particularly abundantly in the new cell walls (Figure 5J, inset). A similar pattern of distribution was observed for the AGP epitopes recognized by the JIM8 and JIM13 antibodies (Figure 5K,L). In the PSK-treated cells the pectic epitope recognized by the LM19 antibody was detected only in the walls resulting from the first division of protoplast-derived cells (Figure 5M), while the pectic epitope recognized by the LM20 antibody in the cytoplasmic compartments (Figure 5N). The AGP epitopes recognized by the JIM8 and JIM13 antibodies presented a similar distribution as described for the control cells (Figure 5O,P). After four days of culture only the AGP epitope recognized by the JIM4 antibody and the extensin epitope did not occur in the walls.

#### 2.2.3. *Daucus Montevidensis*

**Figure 6:**
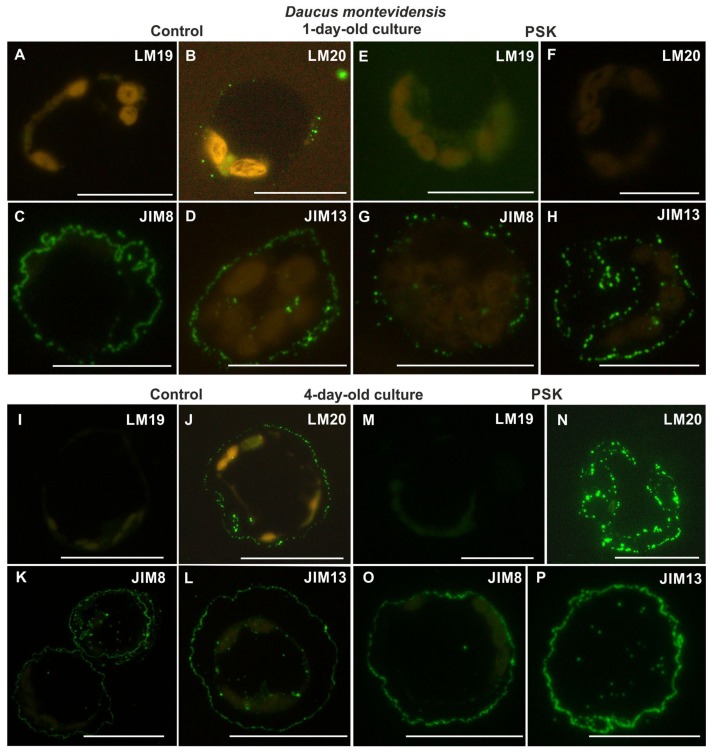
Presence of pectic (LM19, LM20; **A,B,E,F,I,J,M,N**) and AGPs (JIM8, JIM13; **C,D,G,H,K,L,O,P**) epitopes during cell wall regeneration in one- and four-day-old protoplast cultures of *Daucus montevidensis* (scale bars = 10 µm).

From the analyzed pectic epitopes the LM19 was not detected in either one-day-old control or PSK-treated cultures (Figure 6A,E). The LM20 epitope in the control protoplast-derived cells occurred in the form of dotted signal (Figure 6B), while it was not found in the cells from the PSK-treated cultures (Figure 6F). The AGP epitope recognized by the JIM4 antibody was not identified in either the control or the PSK-treated cultures. The AGP epitope recognized by the JIM8 antibody in the control cultures was more abundant than in the PSK variant (Figure 6C,G). In control it was located in the cell wall all over its circumference (Figure 6C), while in the PSK-supplemented cultures the signal was not continuous along the wall circumference (Figure 6G). The AGP epitope recognized by the JIM13 antibody occurred abundantly in both the control and the PSK-treated cultures, although it was more abundant in the PSK variant (Figure 6D,H). The extensin recognized by the JIM12 antibody was detected neither in the control nor in the PSK-treated cultures.

In four-day-old cultures the pectic epitope recognized by the LM19 antibody was not detected in either the control or the PSK variants (Figure 6I,M). The pectic epitope recognized by the LM20 antibody occurred abundantly both in the control and the PSK-treated cultures, with a much more intensive signal in the presence of PSK (Figure 6J,N). The AGP epitopes recognized by the JIM8 and JIM13 antibodies were abundant both in the walls and in the cytoplasmic compartments of the control and the PSK-treated cultures (Figure 6K,O,L,P), while the AGP epitope identified by the JIM4 antibody and the extensin epitope were not detected.

### 2.3. Cell Wall Composition in the Middle Stages of the Culture

#### 2.3.1. *Daucus Carota* Subsp. *sativus*

**Figure 7:**
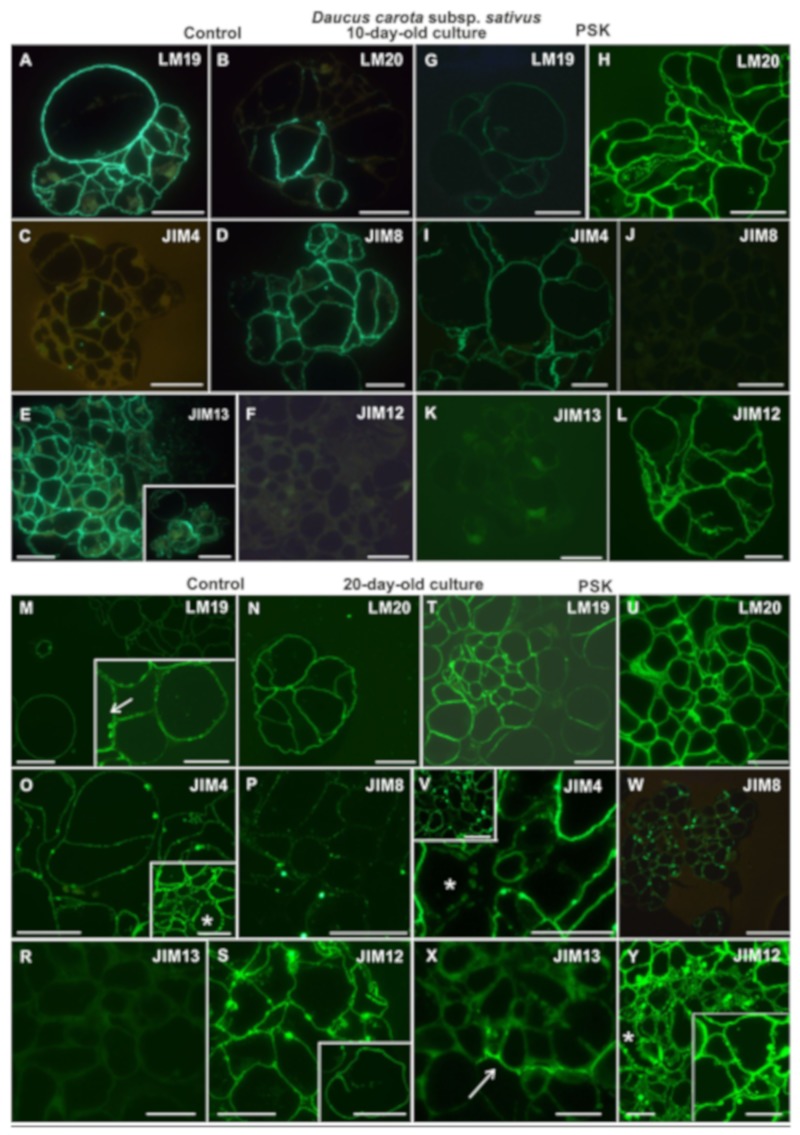
The presence of pectic (LM19, LM20; **A,B,G,H,M,N,T,U**), AGPs (JIM4, JIM8, JIM13; **C,D,E,I,J,K,O,P,R,V,W,X**) and extensin (JIM12; **F,L,S,Y**) epitopes during cell wall regeneration in 10- and 20-day-old protoplast-derived cells of *Daucus carota* subsp. *sativus* (inset on **E** shows the presence of epitope outside the cell wall; insets on **M** (arrow) and **O** (star) show a higher magnification of the tracheary elements; inset in **V**—lower magnification of section, where a star indicates the tracheary element, inset in **S**—fragment of a new wall after first protoplast-derived cell division; inset in **Y**—signal localization in cytoplasmic compartments; scale bars = 20 µm).

In 10-day-old control cultures the pectic epitopes recognized by the LM19 antibody was abundantly present in walls both in the single cells still present in the culture and in the cell complexes, which at that point of culture were multicellular (Figure 7A). In contrast, the LM20 epitope was present mainly in the new cell walls, which is a good marker for the emerging cellular complexes (Figure 7B). In the PSK-treated cells the distribution of these pectic epitopes was diverse in comparison to control cells. Namely, the LM19 epitope was detected in the walls of cell complexes (Figure 7G) while the LM20 epitope was abundantly present in the walls of all cells as well as in the cytoplasmic compartments (Figure 7H). Among the analyzed AGP epitopes there were significant differences between the control and the PSK-treated cultures. The epitope recognized by the JIM4 antibody was detected in the control cells but in a very low amount in the form of separated dots within the walls (Figure 7C), as opposed to the PSK treated cells where this epitope was abundantly present (Figure 7I). In the control cultures the epitope recognized by the JIM8 antibody was frequently detected in all cell walls (Figure 7D) while it was not detected in the PSK-treated cultures (Figure 7J). Similar differences characterized the presence and distribution of the AGP epitope recognized by the JIM13 antibody (Figure 7E and inset, K). The extensin epitope recognized by the JIM12 antibody was not detected in the control cultures (Figure 7F) and was abundantly present in the PSK-treated cultures (Figure 7L).

After 20 days of culture all the analyzed epitopes were detected in the walls of cells both in the control and the PSK-treated cultures (Figure 7) apart from the AGP epitope recognized by the JIM13 antibody, which was not present in the control culture (Figure 7R), and in the PSK-treated cells it was detected only in some cell walls (Figure 7X). The pectic epitope recognized by the LM19 antibody was abundantly present in the walls (Figure 7M,T) and in the control cultures it was present in the walls of differentiating tracheary elements (Figure 7M arrow at inset). The AGP epitope recognized by the JIM4 antibody was a characteristic component of the secondary wall thickenings in the developing tracheary elements (Figure 7O star in the inset, Figure 7V; star). The extensin epitope recognized by the JIM12 antibody in the cells from the control cultures was distributed uniformly in the walls apart from some walls areas, which in most cases represented the cell walls’ corners (Figure 7S). In the newly formed walls this epitope was also detected (Figure 7S inset). In the PSK-treated culture this epitope was present in all the cells, including differentiating tracheary elements (Figure 7Y star) and, apart from the walls, it was also located in the cytoplasmic compartments (Figure 7Y inset).

#### 2.3.2. *Daucus Carota* Subsp. *gadecaei*

**Figure 8:**
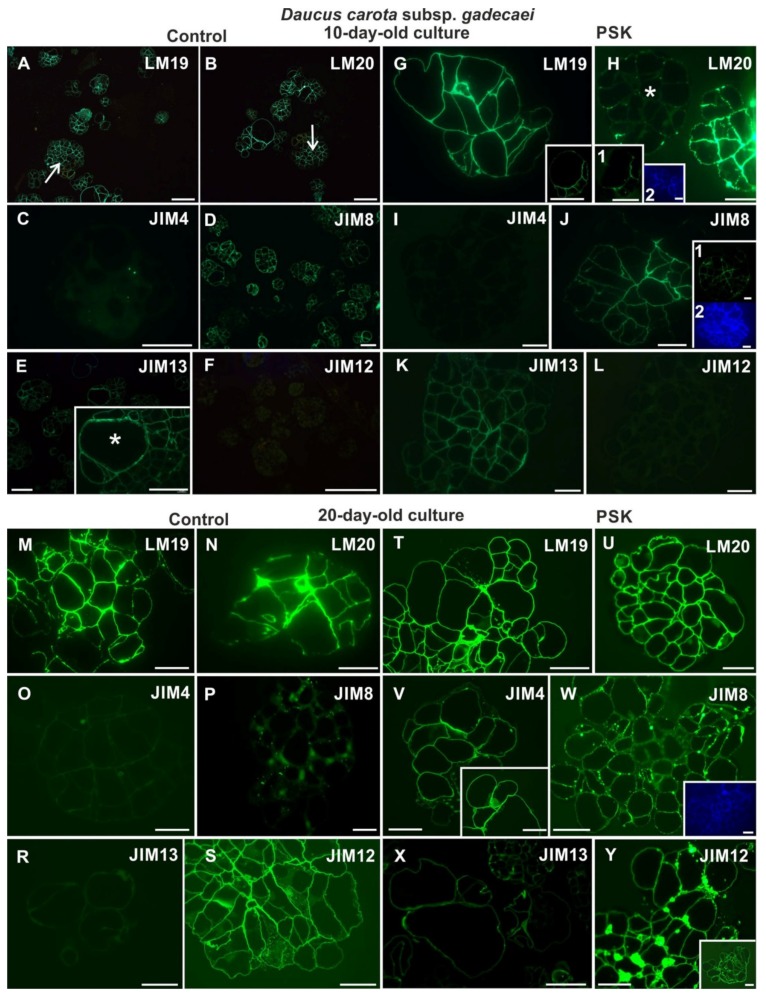
The presence of the pectic (LM19, LM20; **A,B,G,H,M,N,T,U**), AGP (JIM4, JIM8, JIM13; **C,D,E,I,J,K,O,P,R,V,W,X**) and extensin (JIM12; **F,L,S,Y**) epitopes during cell wall regeneration in 10- and 20-day-old protoplast cultures of *Daucus carota* subsp. *gadecaei* (arrows—PEM and/or cell clusters; the inset in **E** shows the abundant presence of epitopes in tracheary elements and the surrounding cells; the inset in **G** is the same part of culture as in the **H** inset 1 showing diverse distribution of the analyzed LM19 and LM20 epitopes; the inset 1 in **J** shows another example of epitope presence in groups of cells; the inset in **V** shows the presence of an epitope in the cytoplasmic compartments; the inset in **Y** shows epitope presence in the walls; the insets in blue in **H**, **J** and **W**—the sections stained with Calcofluor white showing cellulose walls; asterisks—the tracheary element; scale bars = 20 µm).

In 10-day-old cultures the analyzed pectic epitopes were detected in the control and PSK-treated cells (Figure 8A,B,G,H). However, the pectic epitope recognized by the LM19 antibody in the control cultures was present only in new walls (Figure 8A) while in the PSK-variant it was detected in all walls of the cells present in the culture (Figure 8G). In the case of the pectic epitope recognized by the LM20 antibody in the control cultures it was present in the new walls within the cell clusters (Figure 8B) while in the PSK treated cultures, in some cell clusters, this epitope was detected mostly in the inner walls of such clusters, and in others, only in internal walls in the form of separated dots (Figure 8H and insets). Among AGP epitopes the one recognized by the JIM4 antibody was not identified in the PSK-treated cultures but in the control cultures it was detected in very low amount in the form of single dots in the walls inside the cluster (Figure 8C,I) while the AGPs recognized by the JIM8 and JIM13 antibodies were abundantly present (Figure 8D,E,J,K). The differences between the distribution of the AGP epitope recognized by the JIM8 antibody between the control and the PSK-treated cultures lie in the signal presence in most of the walls in cells from the control culture, and in the PSK-treated culture mostly in the walls located inside the cell clusters/PEMs (Figure 8D,J and insets). It appeared that the AGP epitope recognized by the JIM13 antibody is not present in the secondary cell walls (Figure 8E inset). At that time point the extensin recognized by the JIM12 antibody was not detected in either type of culture.

In 20-day-old cultures pectic epitopes were detected in both (the control and the PSK-treated) types of cultures (Figure 8M,N,T,U). The epitope recognized by the LM19 antibody was uniformly located in the walls of cell clusters independent of the culture variant and was also found in the cytoplasmic compartments (Figure 8M,T). In the case of the pectic epitope recognized by LM20 in control cultures this epitope was detected mostly in the internal walls of cell clusters/PEMs, and in the PSK-treated cultures it was also present in the outer walls of cell clusters/PEMs (Figure 8N,U). In the control, the AGP epitopes recognized by the JIM4 and the JIM13 antibodies were not detected (Figure 8O,R), while in the PSK variant they were abundantly present (Figure 8V,X). In the control culture only the AGP epitope recognized by the JIM8 antibody was detected (Figure 8P) whereas the extensin recognized by the JIM12 antibody was present in the cells of each culture (Figure 8S,Y).

#### 2.3.3. *Daucus montevidensis*

**Figure 9:**
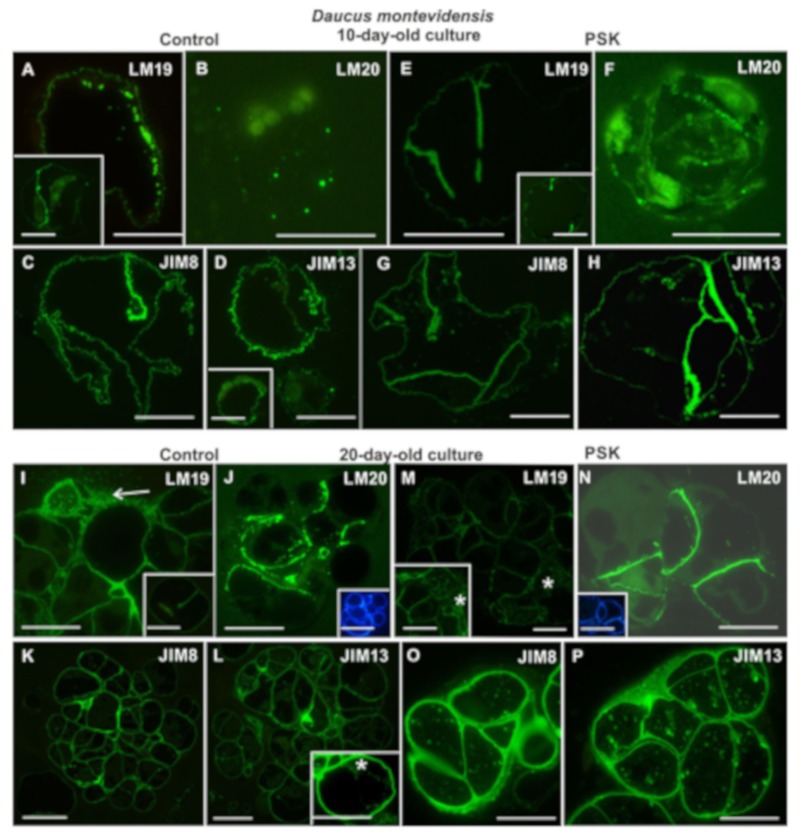
The presence of pectic (LM19, LM20; **A,B,E,F,I,J,M,N**) and AGPs (JIM8, JIM13; **C,D,G,H,K,L,O,P**) epitopes during cell wall regeneration in 10- and 20-day-old protoplast cultures of *Daucus montevidensis* (the insets in **A**, **E** and **I**—the presence of the pectic epitope in the new wall after the first protoplast-derived cell division; the insets in **J** and **N**—the sections stained with Calcofluor white showing the cellulose walls; the arrow in I—extracellular localization of the signal; the inset in **M** and **L**—the presence of signal in cytoplasmic compartments; the asterisk in M and L indicates the cytoplasm; scale bars = 20 µm).

After 10 days of culture the epitope recognized by the LM19 antibody was present both in the control and the PSK-treated variant (Figure 9A,E). At that time point in the culture single cells were still present as well as two-cell aggregates. In single cells, this epitope occurred in the wall and in the cytoplasmic compartments in the vicinity of the wall, both in the control and in the PSK-treated cultures. However, in the dividing cells, it occurred in the new walls, especially in the control cells (Figure 9A and inset; Figure 9E and inset). The pectic epitope recognized by the LM20 antibody was present in the control culture (Figure 9B), but in less amount in comparison to the PSK variant, where this epitope was clearly visible in the new wall after the first division of the protoplast-derived cell (Figure 9F). Within the AGPs epitopes, only those recognized by the JIM8 and JIM13 antibodies appeared (Figure 9C,D,G,H). They were located both in the cell walls, in cytoplasmic the compartments, and outside the wall (Figure 9D inset). The AGP recognized by the JIM4 epitope and the extensin epitope recognized by JIM12 antibody were not detected.

In 20-day-old cultures, the cell complexes composed of a few to several cells were observed. At that time point, pectic epitopes were abundantly present in the control and the PSK-treated cultures (Figure 9I,J,M,N). The pectic epitope recognized by the LM19 antibody, apart from being present in the walls, was secreted outside the cells (Figure 9I arrow) and was the main component of the new walls (Figure 9I inset; Figure 9M and inset). The location of the pectic epitope recognized by the LM20 antibody was very interesting as it occurred only in the walls inside the cell complexes, and these walls were clearly new walls after the first protoplast-derived cell division (Figure 9J,N; the insets indicate cellulose in the same cell complexes). The AGP epitopes recognized by the JIM8 and JIM13 antibodies were present both in the cell walls and in the cytoplasmic compartments, which indicates intensive synthesis of these epitopes by the cells (Figure 9K,L,O,P). Similarly, as in 10-day-old cultures, the epitopes detected by the JIM4 and JIM12 antibodies did not occur. 

### 2.4. Distribution of Pectic, AGP and Extensin Epitopes in Mature Cultures

#### 2.4.1. *Daucus Carota* Subsp. *sativus*

**Figure 10:**
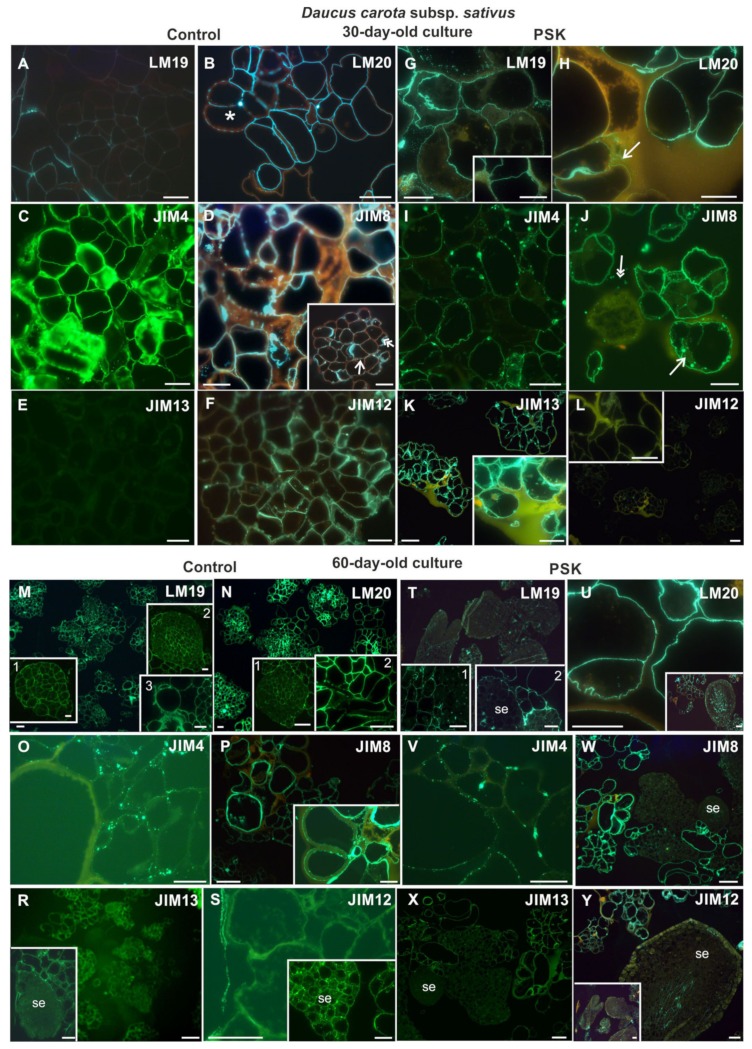
The presence of pectic (LM19, LM20; **A,B,G,H,M,N,T,U**), AGP (JIM4, JIM8, JIM13; **C,D,E,I,J,K,O,P**) and extensin (JIM12; **F,L,S,Y**) epitopes during cell wall regeneration in 30- and 60-day-old protoplast cultures of *Daucus carota* subsp. *sativus* (inset in **D**: the double arrow points to the epitope presence in cytoplasmic compartments and the single arrow points to the epitope presence in the wall; inset in **G**—higher magnification of the presence of the epitope in the cell wall; insets in **K** and **L** show higher magnification of cell complexes; insets in **M**: inset 1—PEM; inset 2—somatic embryo; inset 3—the signal outside the wall; insets in **N**: inset 1—PEM; inset 2—tracheary elements; insets in **T**: inset 1—PEM, inset 2—somatic embryo (se); inset in **U**—somatic embryo; inset in **P**—tracheary elements; insets in **R**, **S** and **Y** show somatic embryos (se) at different stages of development; the double arrow—epitope localization outside the wall, arrow—in the cell wall; scale bars = 20 µm).

In 30-day-old cultures pectic epitopes were detected in both culture types (Figure 10A,B,G,H). In the control, the pectic epitope recognized by the LM19 antibody was present only in the cell walls (Figure 10A), while in the PSK-treated cells it was detected also in the cytoplasmic compartments (Figure 10G). The epitope recognized by the LM20 antibody in the PSK variant, apart the walls, was also detected outside the cell complexes (Figure 10H arrow). In the control cultures it is visible that this epitope is present only in the primary cell walls (Figure 10B). The AGP epitopes recognized by the JIM4 and JIM8 antibodies were detected both in the control and the PSK-treated cultures (Figure 10C,D,I,J). The analysis of the control cultures has shown abundant presence of the AGP epitope recognized by the JIM8 antibody in the outer walls of cell clusters and in the walls on the borders with the intercellular spaces (Figure 10D and inset). In the PSK-treated culture, apart from walls, this epitope was also detected in cytoplasmic compartments and outside the walls (Figure 10J). The AGP epitope recognized by the JIM13 antibody was not detected in the control cultures (Figure 10E), however, it was abundantly present in the walls of the cell complexes in the PSK-treated variants (Figure 10K). The extensin recognized by the JIM12 antibody was detected only in the control (Figure 10F,L).

At the end of the culture period in alginate matrix (i.e., in 60-day-old cultures) the pectic epitopes recognized by the LM19 and LM20 antibodies were present in the walls of cells from each type of culture (Figure 10M,N,T,U). In the control cultures, the pectic epitope recognized by the LM19 antibody was abundantly present in cell clusters, and in somatic embryos at the globular stage of development it was present in the walls of all embryo cells (Figure 10M inset 1) but at older stages this epitope was detected in the developing provascular tissue (Figure 10M inset 2). Moreover, the abundant presence of this epitope was detected extracellularly (Figure 10M inset 3). The same epitope in the PSK-treated cultures was not detected in somatic embryos but was present in cell clusters (Figure 10T). In the control cultures the pectic epitope recognized by the LM20 antibody was present abundantly in cell clusters (Figure 10N inset 2) and in the walls of somatic embryos located inside the embryo marking the position of provascular tissue (Figure 10N inset 1). In the PSK-treated cultures the same epitope was detected in the walls of cell clusters (Figure 10U) and in somatic embryos it was present in protodermal cells and the provascular tissue (Figure 10U inset). AGP epitopes were present in the walls of cells independent of the culture type (Figure 10O,P,R,V,W,X), however it was never detected in cell clusters or somatic embryos (Figure 10R,W,X). The AGP epitope recognized by the JIM4 antibody was abundantly present in cell clusters both in the control and the PSK-treated cultures (Figure 10O,V). The signal generated by the JIM8 antibody was uniform along the wall circumference (Figure 10P,W), and the signal generated by the JIM13 antibody was less intensive in the control (Figure 10R) in comparison to the PSK-treated cultures (Figure 10X). The extensin detected by the JIM12 antibody was almost absent from the control cultures apart from certain structures, where it was located outside the cell complexes (Figure 10S), but it was abundantly present in somatic embryos (Figure 10S inset). In the PSK-supplemented cultures this epitope was present in the cell walls, and in the provascular tissue of somatic embryos (Figure 10Y inset).

#### 2.4.2. *Daucus Carota* Subsp. *gadecaei*

**Figure 11:**
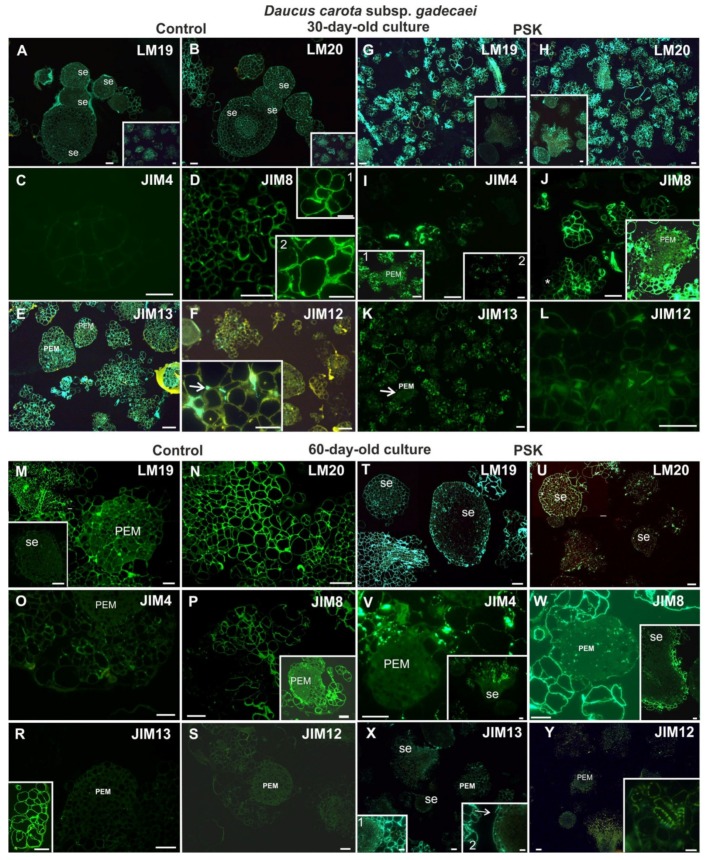
The presence of pectic (LM19, LM20; **A,B,G,H,M,N,T,U**), AGP (JIM4, JIM8, JIM13; **C,D,E,I,J,K,O,P,R,V,W,X**) and extensin (JIM12; **F,L,S,Y**) epitopes during cell wall regeneration in 30- and 60-day-old protoplast cultures of *Daucus carota* subsp. *gadecaei* (insets in **A** and **B**—the larger area of the culture; insets in **D** higher magnification of cell walls; the inset in **F** shows the presence of the epitope in the extracellular spaces; the insets in **G** and **H**—somatic embryos at different stages of development; the inset in **I**: 1—PEM and the surrounding cell clusters, 2—the presence of the epitope in the walls of cell clusters; the inset in **J**—PEM and the surrounding cells; the inset in **M**—a somatic embryo; the inset in **P**—the lack of signal in PEM; the insets in **V**, **W** and **X**—signal localization in somatic embryos at different developmental stages; in X: inset 1 border between se and cell clusters, inset 2—protodermal cells of se (arrow); PEM—proembryogenic mass; se—somatic embryos; scale bars = 20 µm).

Thirty-day-old cultures were characterized by the presence of pectic epitopes both in the control and the PSK variant (Figure 11A,B,G,H). In the control cultures, the pectic epitope recognized by the LM19 antibody in the cell clusters and PEMs was present inside these structures (Figure 11A inset). In somatic embryos it was not detected but it was visible in the extracellular matrix covering the embryos (Figure 11A). In the PSK treated cultures this epitope was present abundantly in the cell clusters (Figure 11G). In somatic embryos, the distribution of this epitope was similar to the control culture (Figure 11G). In the control cultures, the pectic epitope recognized by the LM20 antibody was present in the walls of somatic embryos with a higher level in the provascular tissue (Figure 11B). In the PSK-treated cultures this epitope was detected in the cell clusters and PEMs (Figure 11H) while in the somatic embryos it was present in a lesser extent in comparison to the control culture and it was not abundantly present in the provascular tissue (Figure 11H and inset). The AGP epitope recognized by the JIM4 antibody in the cells from the control cultures was not detected (Figure 11C) but in the PSK-treated cultures it was present in some cell clusters (Figure 11I and inset 2) but never in the PEMs (Figure 11I inset 1). The AGP epitope recognized by the JIM8 antibody was abundantly present in the walls of cell clusters both from the control and the PSK-treated cultures (Figure 11D, J) but it was never detected in PEMs or in differentiating tracheary elements (Figure 11J and insets). In the control cultures, an abundant presence of this epitope was detected in the extracellular matrix (Figure 11D and insets). The AGP epitope recognized by the JIM13 antibody was detected in both culture types, but in the PSK-treated cells it was not detected in PEM (Figure 11K arrow). The extensin epitope was recognized in both types of culture but in much lower amount in comparison to younger cultures (Figure 11F, L).

In 60-day-old cultures, the pectic epitopes recognized by the LM19 antibody was detected in the walls of cultured cells independent on the culture type including PEMs and cell clusters (Figure 11M,T). In the control cultures, this epitope was hardly detected in very young somatic embryos (Figure 11M inset) and in the PSK-treated cultures the older stage of somatic embryo development the less amount of this epitope in walls (Figure 11T). The pectic epitope recognized by the LM20 antibody was abundantly present in the cells, cell clusters and somatic embryos independent of the culture type (Figure 11N,U). AGP epitopes were detected in both types of culture (Figure 11O,P,R,V,W,X) and in each case these epitopes were not detected in PEM and somatic embryos apart from the AGP epitope recognized by the JIM13 antibody which was detected in the surface layers of somatic embryos (Figure 11X). The extensin recognized by the JIM12 antibody was not detected, apart from a secondary thickening of the tracheary elements walls in the PSK-treated cultures (Figure 11S,Y and inset).

**Figure 12:**
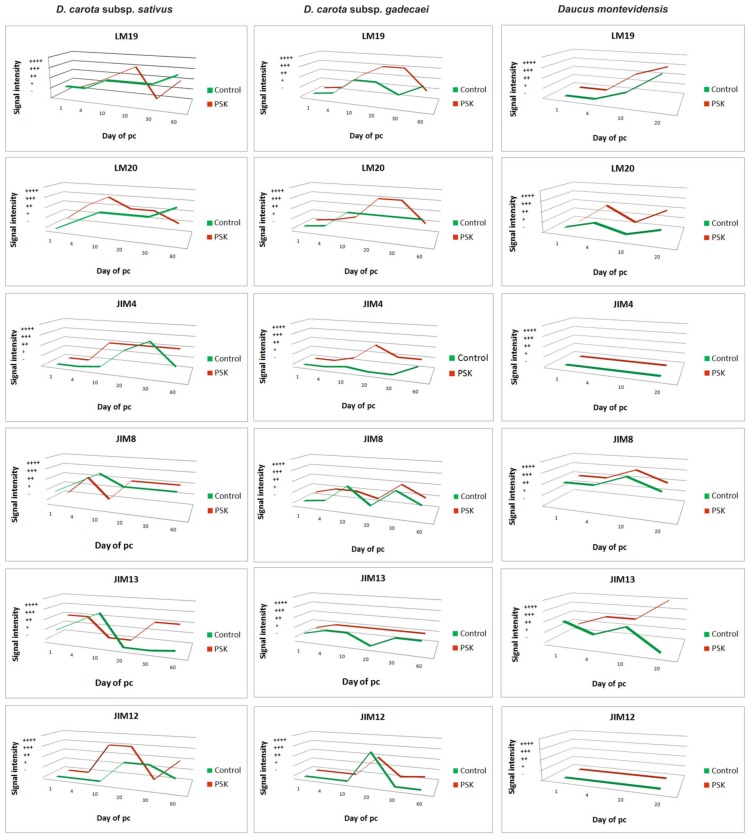
A summary of the presence of pectic (LM19, LM20), AGP (JIM4, JIM8, JIM13) and extensin (JIM12) epitopes during cell wall regeneration in the control and the PSK-treated protoplast cultures (pc) of three *Daucus* taxa.

Quantitative and qualitative differences were found between the studied taxa as well as between the culture period and PSK treatment. It seems that there is variation depending on the species, the analyzed epitopes are developmentally regulated and that such regulation may be modified in the presence of PSK. The detected differences in the chemical composition of the pectic, AGP and extensin epitopes in cells during the wall regeneration may explain the degeneration of the culture and the stimulation of embryological competence by PSK during the culture development and cell wall regeneration. The lack of AGP and extensin epitopes recognized respectively by the JIM4 and JIM12 antibody in *Daucus montevidensis* protoplast cultures may be the reason (among others) for the degeneration of those cultures. In all of the analyzed time variants of the experiment, it was found that the presence of the studied epitopes between 10–20 days of the culture was increased (Figure 12). In the PSK-treated cultures, the pectic epitopes recognized by the LM19 and LM20 antibodies in *D. carota* subsp. *gadecaei* appeared from the first day of culture, i.e., several days earlier in comparison to the control (Figure 12). Similar results were obtained for *D. carota* subsp. *sativus* for the pectic epitope recognized by the LM20 antibody (Figure 12). In the case of all analyzed AGPs, PSK increased their amount in the culture. The AGP recognized by the JIM8 antibody was found in the walls of the cells throughout the entire PSK-supplemented cultures, while in the control they disappeared about the 20th day of culture, which may strongly suggest that PSK stimulates their synthesis. The amount of the extensin recognized by the JIM12 antibody was higher in the PSK-treated cultures and in *D. carota* subsp. *gadecaei* it was detected throughout the entire culture period while in the control cultures it disappeared about the 30th day of culture.

### 2.5. Summarizing

(a) Among the analyzed taxa the *D. montevidensis* expressed strong recalcitrance to the culture condition independent of the culture type (control or PSK-treatment) and degenerated after 20 days of culture, which might be correlated with the lack of synthesis of the AGP and extensin recognized by the JIM4 and JIM 12 antibody, respectively.

(b) *Daucus carota* subsp. *sativus* in comparison to *Daucus carota* subsp. *gadecaei* had: (a) a higher level of pectins (LM19, LM20); (b) a similar level of the AGPs recognized by the JIM4 and JIM8 antibody; (c) a lower level of the AGP recognized by the JIM13 antibody and (d) a higher level of the extensin recognized by the JIM12 antibody.

(c) The cells of *Daucus carota* subsp. *sativus* derived from the PSK-treated cultures in comparison to control showed: (a) the lack of any differences in pectic and AGP epitopes after 10 days of culture, (b) differences in AGP epitope localization recognized by the JIM4 antibody after 20 days of culture and (c) abundant presence of extensin after 60 days of culture.

(d) The cells of *Daucus carota* subsp. *gadecaei* derived from the PSK-treated cultures in comparison to the control cultures were characterized by: (a) a lower presence of the pectic epitope recognized by the LM19 antibody, (b) changes in the presence of the pectic epitope recognized by the LM20 antibody in respect to culture duration and (c) the presence of AGP the epitopes and the extensin recognized by the JIM12 antibody.

## 3. Discussion

Protoplast cultures have a wide range of applications, from the possibility of obtaining whole plants, somatic hybrids, performing single cell cloning and genetic transformations to being an excellent material for cellular and ultrastructural studies. Although, over the years, many systems of protoplast culture have been applied to hundreds of species, especially to a number of cultivated species, the regeneration of protoplasts is occasional or its efficiency is low [7,17,32,33,34]. Somatic hybridization as a practical application of protoplast-based systems is a desirable alternative to conventional crop breeding allowing not only the introduction of unique traits from wild forms into the available pool of breeding materials but also it also significantly reduces the time required for their development. Despite the fact that the carrot is a model plant for in vitro cultures, there are not many reports on its intra-/interspecific as well as intergeneric somatic hybridization and so far the only species within the genus *Daucus* explored in this context, has been *D. carota* subsp. *capillifolius* [12,35,36,37]. Moreover, beside the carrot not many other *Daucus* species have been exploited with respect to developing a successful protoplast-to-plant system. To the best of our knowledge, until now, six *Daucus* accessions including three wild subspecies of *D. carota* (*D. carota* subsp. *azoricus, D. carota* subsp. *drepanensis* and *D. carota* subsp. *maritimus*) and three wild *Daucus* species (*D. aureus*, *D. pusillus* and *D. montevidensis*) have been evaluated for their totipotency in protoplast cultures [16]. Among them, two species, i.e., *D. montevidensis* and *D. pusillus*, expressed strong recalcitrance to culture conditions by the arrest of cell divisions after only a few rounds of mitosis, while the remaining four wild forms were successfully regenerated with efficiencies not lower than those observed for the cultivated carrot. Such diverse response of the wild relatives in comparison to the cultivated crop to the same culture conditions is a known phenomenon [32]. In that context, the *Daucus* species seems to be an interesting model system to study the process of protoplast and protoplast-derived cells redifferentiation, especially in early and thus critical stages of the culture.

The analysis of the cell wall components including some pectic, AGPs and extensin epitopes during the protoplast wall regeneration with the use of monoclonal antibodies (mAbs) has not been performed for the carrot (at least to the best knowledge of the authors), which confirms that the specific spatio-temporal analysis of changes in the wall composition of carrot protoplast-derived cells is fully justified. As the object of our studies we have chosen three *Daucus* taxa with diverse responses to in vitro cultures. The protoplast yield, their quality (evaluated with fluorescein diacetate (FDA) staining) and the level of protoplast development were comparable to the results observed previously [16] proving that isolation and the culture system developed by us for the cultivated carrot [15] is highly repeatable.

It is known that the chemical composition of cell walls changes during plant growth and development and that there is a correlation between cell differentiation and cell wall chemistry [21,38,39,40]. Cell wall composition is also an important factor controlling plant regeneration from protoplast cultures [19,22,29]. It is postulated that the cell wall reconstruction is a key step in reprogramming and dedifferentiation in protoplast cultures [19,41]. Diverse cell wall constituencies are postulated to regulate the de- and redifferentiation processes during protoplast wall re-synthesis.

During wall regeneration, what is important is not only the wall composition, but also the distribution of the components within the re-synthesized walls, which is possible to determine only using immunohistochemical assays with mAbs directed to specific wall epitopes. Such studies have been performed only in a few (at least to the best of our knowledge) species such as *B. vulgaris* and *N. tabacum* [19,28,42]. Therefore, we believe that the obtained results increase the knowledge of the participation of various epitopes of pectins, AGPs and extensins in the process of cell wall regeneration especially that they refer to the species of great agronomic importance. In addition, a comparison of the temporal and spatial changes in the composition of re-synthetized wall between the control and the PSK-treated cultures may have an impact on the use of this peptidyl plant growth factor in increasing the efficiency of protoplast-to-plant systems.

### 3.1. Distribution of Pectins with Different Levels of Esterification Change in a Diverse Manner Depends on the Culture Condition and the Species

The LM19 HG domain in pectic polysaccharides recognizes a range of HG with the preference to bind strongly to unesterified HG, while the LM20 HG domain requires methyl esters for recognition of HG and does not bind to unesterified HG [43]. It is known that HG is exported to the cell wall in a fully methyl-esterified form and changes in the HG esterification are a result of the pectin methyl-esterase activity (PME; [44]), which means that the analysis of these two epitopes allows us not only to determine their presence and distribution within regenerated walls, but indirectly allows us to make suppositions about the PME activity.

The obtained results showed that the pectic epitopes recognized by the LM19 and LM20 antibodies were not present after one day of culture apart from the *D. montevidensis* where esterified pectins were detected, however in a small amount. After 4 days of culture, esterified pectins were abundantly present in the walls, especially in the PSK-treated protoplasts. Along with the prolongation of the culture age the amount of both non-esterified and esterified pectins increased, regardless of the species, but the amount in the PSK-treated cultures was higher in comparison to the control, especially in the middle-aged cultures. This indicates that during the wall regeneration the esterified pectins are deposited first, which is in accordance with other data [38,44]. In general, the obtained results are difficult to compare with literature because very little data relates to the recovery of pectins during the reconstitution of the wall in protoplast-derived cells, even less applies to the synthesis and distribution of esterified and non-esterified pectins and still less provides information on the molecular level with the use of mAbs directed to specific chemical components of the wall during regeneration. Abundant presence of the methyl-esterified pectin recognized by the JIM7 antibody was detected in olive protoplast cultures [42]. It is known that in *Daucus carota* L. subsp. *sativus* protoplasts-derived cells, the amount of carbohydrate increased significantly with only minor changes in neutral sugar composition, however, detailed information about the presence of esterified and non-esterified pectins was not provided [45]. In the *Lilium longiflorum* pollen-derived protoplasts the first components deposited in the new wall were highly esterified pectins while low esterified pectins were not detected (at least in 3-day-old cultures; [46]). In beet and tobacco protoplast cultures a widespread occurrence of the epitope recognized by the LM6 antibody, which binds to the (1–5)-α-l-arabinans RG I side chain, was detected [29].

Cell wall flexibility and elasticity depends on the degree of pectin esterification, facilitating cell adaptation to the changes in volume [42]. Thus, the presence of this type of pectin in carrot protoplasts-derived cells during wall regeneration undoubtedly adapts cells to the changing external conditions. Methyl-esterified pectic polysaccharides were also reported to form the wall of actively growing somatic protoplasts and somatic cells in vitro [47,48]. The detected correlation, presented in the data here, between the amount of esterified pectins and PSK may indicate that this peptidyl plant growth factor has a positive impact on the synthesis and deposition of pectins during wall regeneration, which is consistent with our observations indicating a positive influence of PSK on the culture efficiency. Moreover, it was proposed that de-esterification of pectins permits the formation of a gel that envelops the protoplasts allowing rigid cellulose–hemicellulose framework formation along with this gel matrix [49]. It is possible that PSK influences the response of the protoplasts-derived cells also on this level.

Based on the analysis of the expression profile of PSK genes, it may be thought that phytosulfokines participate in the regulation of cell division and cell de-differentiation in somatic embryogenesis as well as in the formation of adventitious roots [30,50,51]. The analysis of the hypocotyl-derived protoplasts of *Arabidopsis* indicated that PSK signaling controls osmotically-driven cell expansion [52]. The results presented here may suggest that PSK is involved also in the process of protoplast wall regeneration, which would be in accordance with the research, which highlights the role of *AtPSK* in the regulation of cell size [53]. However, to confirm this supposition, further research on the molecular level is required.

### 3.2. Expression and Localization of AGPs in Protoplast-Derived Cells and Cell Aggregates are Time and Tissue Specific and Enhanced by PSK

AGPs are involved in diverse developmental processes such as plant reproduction [54], pattern formation [55,56,57], adventitious root development [58], somatic and zygotic embryogenesis [21,27,54,59,60], cell division [61], expansion [62] and cell death [27,57]. We studied the distribution of the epitopes during wall regeneration of carrot protoplasts and in the protoplast-derived cell aggregates that were recognized by the JIM4, JIM8 and JIM13 antibodies. Based on such a selection we were able to compare our results with the only little data available in the literature referring to sugar beet and tobacco protoplasts [19,54,63,64].

The AGP epitope recognized by the JIM4 antibody was not detected in *D. montevidensis* protoplast-derived cells while it was easily identified in the carrot and the *D. carota* subsp. *gadecaei* protoplast-derived cells. In both cases, in the PSK-treated cultures, the level of this epitope was higher in comparison to the control. Two other AGPs epitopes recognized by the JIM8 and JIM13 antibodies were detected in protoplast-derived cells and cell aggregates with changing level during the culture period but always in a higher amount in the PSK-treated cultures. So far there is little existing data describing the presence and changes of the AGPs in the protoplast cultures and even less referring to the immunolocalization of different AGPs epitopes. In the regenerating protoplasts of the beet and the tobacco, widespread occurrence of epitope recognized by anti-AGP JIM13 antibody was detected, while the epitope reacting with the JIM4 antibodies was not localized [29]. In sugar beet protoplast cultures the signal intensity generated by the JIM8 and JIM13 antibodies varied between individual cells of the same population and in successive stages of development [28], which is similar to the results obtained for the carrot in the present studies. The JIM8- and JIM13-responsive epitopes were widespread in sugar beet cells of different origin and at different stages of cell wall synthesis, which suggests that these epitopes may play an important role in cell wall formation and growth under in vitro conditions. The data obtained here for the *Daucus* protoplast-derived cells are in accordance with the abovementioned results.

The localization of AGP in the plasma membrane, cell walls and extracellular spaces may indicate their role in the wall extensibility, cell wall patterning, cell-cell interaction including adhesion processes and signal transduction pathways [28,61,63,65,66,67]. The obtained results for the carrot are in accordance with literature data as, at least for the early stages of protoplast culture, the JIM8 and JIM13 antibodies were detected not only in the plasma membrane but also outside the regenerating protoplasts. Moreover, the detection of this signal within the cytoplasmic compartments indicates that the synthesis took place and was intensive during that time, similar to the sugar beet [28].

The JIM8 antibody was detected in trace amounts in the regenerating leaf-derived protoplasts of *Beta vulgaris* and not detected in *Nicotiana tabacum* cultures, while the JIM13 antibody was abundantly present in both types of protoplasts [29]. The differences identified by us in the expression of the analyzed AGP epitopes in different carrot species are in accordance with the view that in some species cell differentiation is accompanied by disappearance of definite epitope(s) [21,58,68], whereas in other models, cell specialization occurs concomitantly with the enhanced expression of the same AGP epitope(s) [26,27,69] indicating that AGPs expression is a cell-, tissue- or species specific. The resynthesis of cell walls and extracellular material during the regeneration of the carrot (*Daucus carota* subsp. *sativus*) protoplasts showed an increase in the arabinogalactan proteins level parallel to the formation of cell walls [45]. In the *Marchantia polymorpha* protoplast-derived cells, an immunolabeling study indicated the presence of epitopes recognized by the JIM13 anti-AGP antibodies in the regenerated cell wall and cell plate, which suggests that AGPs have a significant role in the cell plate as well as in the cell wall formation [70]. The presence of these epitopes in cell plates detected in the carrot may indicate that these epitopes play a universal role in the regeneration of protoplasts, which of course needs further studies with different species.

### 3.3. The Increase in the Extensin Level is Characteristic for Middle-Aged Cultures

Extensins belong to the hydroxyproline-rich glycoproteins (HRGPs) superfamily, which is moderately glycosylated [71]. We investigated the distribution of only one epitope, recognized by the JIM12 antibody, which recognizes the unknown extensin epitope [72]. The data obtained here showed that this extensin epitope appeared between the 10th and 20th day of culture, localizing mostly in the provascular tissue of developing somatic embryos and disappeared at the end of culture in alginate matrix (i.e., in 60-day-old-cultures). So far the presence and distribution of this extensin has not been examined during the protoplast wall re-synthesis. The only information concerning the protoplasts showed the extensin gene expression in freshly isolated protoplasts of *Nicotiana sylvestris* [73]. Other information about the presence of extensins, especially recognized by the JIM12 antibody, comes from studies on different developmental processes in various plant species. The analysis of *Daucus carota*, *Raphanus sativus*, *Pisum sativum* and *Allium cepa* showed the presence of the JIM12 antibody in the developing xylem cells in all four species. In carrot roots, JIM12 bound to the metaxylem cells and the regions of the cell wall that line the intercellular spaces [74]. Thus, the results obtained by us are in accordance with the previously published ones but they also increase our knowledge on the spatio-temporal changes in the presence of extensin during the wall regeneration of protoplasts and protoplast-derived cell aggregates not only in the carrot but also in other *Daucus* species. 

It was once assumed that extensins had a role in cell extension. That role is no longer considered valid. Although the precise function of extensins is still unclear, they are now believed to play a stabilizing or reinforcing role in the wall of cells that have stopped elongating [71]. However, to answer the question of the role of extensin in wall regeneration further studies are needed. 

### 3.4. PSK and Wall Modification on the Molecular Level

It is suggested that PSK triggers a signaling cascade, which results in cell wall loosening, as well as turgor pressure-driven protoplast expansion with the involvement of expansins, which are secretory proteins capable of loosening the cell wall network by disrupting non-covalent bonds between polysaccharide chains [75]. Expansins are good candidates for cell wall modifiers, as shown in *Arabidopsis* [76]. To date, it is the only example of the link between PSK and cell wall chemical components. It has been shown recently that RLP44 (receptor-like protein) is a component of the cell wall signaling pathway, joining the exchange of signals from the cell wall to the cytoplasm via interaction with BRI1 (plasma membrane-bound receptor) and BAK1 (receptor-like kinase) [77,78,79]. Thereby, the brassinosteroid (BR) signaling pathway is activated, leading to expression of cell wall biosynthetic and remodeling genes [77]. Additionally, the association of RLP44 and the cell wall was revealed in vitro and in vivo [78]. However, according to Gómez [80], RLP44 interacts with the PSK receptor, supporting the idea that RLP44 shifts the interaction with BRI1 and PSKR1 (phytosulfokine receptor). Thus, RLP44 might integrate the changes detected at the level of the cell wall into two independent signaling pathways and modulate different and/or combined responses, although the nature of the signal perceived by RLP44 is still unknown. Moreover, binding of PSK to the receptor requires acidic conditions, indicating that cell wall acidification increases binding of PSK to the receptor [81]. Thus, presence of pectic epitopes may indicate that such a relationship exists during the cell wall reconstruction in PSK-treated cultures.

There is currently no information about a relationship between PSK and the chemical composition of the cell wall. However, recent data indicate a link between brassinosteroid signaling and the cell wall [77], and between the BR signaling pathway and PSK [78]. Therefore, it can be implicitly concluded that the relationships reported here between PSK and chemical components of the wall exist. Nevertheless, the determination of individual elements of this signal transduction pathway requires further research. At present, the results presented here are the first to reveal such a relationship.

## 4. Materials and Methods

### 4.1. Plant Material

The cells and tissues under investigation were obtained from protoplast cultures of three *Daucus* taxa including the carrot (*D. carota* subsp. *sativus*) cv. Dolanka, *Daucus carota* subsp. *gadecaei* (Rouy and E. G. Camus) Heywood and Daucus montevidensis Link (Table 2). The seeds of all accessions were disinfected according to the procedure described by Maćkowska et al. [16] and placed on the Murashige and Skoog (MS) medium [82], supplemented with 30 g·L^−1^ sucrose (POCH, Gliwice, Poland) and maintained at 18 ± 2 °C in the dark for germination. Seven-day-old seedlings were transferred to glass jars with regeneration (R) medium [16], and kept in a climate room at 26 ± 2 °C under a 16-h photoperiod with light intensity of 55 μmoL·m^−2^·s^−1^.

### 4.2. Protoplast Isolation

Protoplasts were isolated from 2-week-old in vitro grown plants, following the procedure described by Grzebelus et al. [15]. Briefly, leaves were cut into pieces, pre-treated in a plasmolysis solution and then incubated overnight in an enzyme solution for ~16 h on a gyratory shaker (30 rpm; Heidolph Instruments, Schwabach, Germany) at 26 ± 2 °C in the dark. Then the protoplasts were separated from the undigested tissues by filtration through a nylon mesh and purified by sugar gradient centrifugation.

### 4.3. Protoplast Embedding in Alginate Matrix and Culture

Protoplasts at the density 8 × 10^5^ cells per mL were embedded in a 2.8% filter-sterilized sodium alginate (Sigma-Aldrich, St. Louis, MO, USA) solution using the thin alginate layer (TAL) system according to Maćkowska et al. [16]. After alginate gelation, the layers with embedded protoplasts were transferred to 6 cm Petri dishes containing 4 mL of carrot petiole protoplast (CPP) medium [16] or CPP medium supplemented with 100 nM of PSK (phytosulfokine-α, PeptaNova GmbH, Germany). In order to minimize the growth of endogenous bacteria, cefotaxime (Polfa Tarchomin SA, Warszawa, Poland) was applied at a concentration of 400 mg·L^−1^. The cultures were incubated at 26 ± 2 °C in the dark. After 10 days of culture, to remove the toxic products of metabolism, the medium was renewed with a fresh one with all the supplements, as mentioned above.

### 4.4. Evaluation of Culture Development and Data Analysis

To characterize (1) the quality of source tissue and maceration conditions, (2) the quality of released protoplasts and (3) the progress of protoplast development, three basic parameters including the protoplast yield, protoplast viability and plating efficiency were evaluated, respectively. The protoplast yield, expressed as a number of protoplasts per gram of fresh weight (FW) of leaf tissue, was determined using the Fuchs Rosenthal counting chamber. The viability of embedded protoplasts was assessed by staining the cultured cells immediately after immobilization in the alginate, with fluorescein diacetate (FDA; Sigma-Aldrich) according to the procedure by Anthony et al. [83] and Grzebelus et al. [15], and presented in percentages. The plating efficiency expressed as a number of cell aggregates per total number of observed undivided cells and cell aggregates (×100) was evaluated on the 10th, 20th and 40th day of culture. All vital microscopic observations were performed under an Axiovert S100 microscope (Carl Zeiss, Göttingen, Germany) equipped with a filter set appropriate for detecting fluorescein fluorescence (λ_Ex_ = 485 nm, λ_Em_ = 515 nm).

The protoplast cultures were conducted in complete randomized design (CRD) with at least three replicates, where each replicate was an independent round of protoplast isolation. Each treatment was represented by three Petri dishes and about 200 cells per Petri dish were observed. The overall effect of treatments was assessed using the analysis of variance (ANOVA) in Statistica ver. 10.0 (StatSoft. Inc., Poland). Tukey’s honestly significant difference (HDS) test or the least significance test (LSD) was used for mean separation at least at *p* ≤ 0.05.

### 4.5. Sampling, Fixation and Embeding of Plant Materials

The samples were collected at several time-points of the culture i.e., immediately after protoplast isolation, daily from one to five-day-old cultures and from 10-, 20-, 30- and 60-day old cultures (Table 2) both in the control and the PSK-treated variants. Before fixation, the protoplasts or protoplast-derived cell aggregates/tissues were released from the alginate matrix by its depolymerization in a sodium citrate solution [16] and positioned in 1% Seaplaque Agarose (Duchefa Biochemie, Haarlem, The Netherlands) dissolved in phosphate-buffered saline (PBS) consisting of 137 mM NaCl, 2.7 mM KCl, 10 mM Na_2_HPO_4_ and 1.76 mM KH_2_PO_4_ (all from Sigma-Aldrich). The further procedure was carried out according to Potocka et al. [21]. In brief, the samples were fixed in a mixture of 4% formaldehyde (Sigma-Aldrich), 1% glutaraldehyde (Sigma-Aldrich) in PBS at pH 7.2 for 24 h at 4 °C, washed in PBS (pH 7.2), dehydrated in a graded ethanol series (10%, 30%, 50%, 70%, 90% and 100%; *v/v*), infiltrated in LR white resin (medium grade, Polysciences, Hirschberg an der Bergstrasse, Germany) and embedded in gelatine capsules (Agar Scientific, Essex, United Kingdom) with fresh LR white resin and polymerized for 8 h at 50 °C. Semi-thin sections (0.5–1 µm) were cut using a Leica EM UC6 ultramicrotome (Leica Microsystems, Wetzlar, Germany) and mounted on poly-L-lysine coated microscope slides (Menzel-Glaser, Braunschweig, Germany).

### 4.6. Immunological Assay

The sections were blocked in the blocking buffer containing 2% fetal bovine serum (Sigma) and 2% bovine serum albumin (Jackson ImmunoResearch Laboratories, UK) in PBS for 30 min and incubated with primary monoclonal antibodies (diluted 1:10) at 4 °C overnight. All of the primary antibodies used in the study were purchased from PlantProbes (Leeds, UK) and are listed in Table 3. After washing in the blocking buffer (5 × 10 min), the sections were incubated with the secondary antibody (Alexa Fluor 488-conjugated AffiniPure goat anti-rat IgG H+L, Jackson ImmunoResearch Laboratories) diluted 1:100 in the same buffer for 1 h at room temperature. Finally, the slides were washed in the blocking buffer (5 × 10 min), rinsed in PBS followed by sterile distilled water and mounted with a Fluoromount aqueous mounting medium (Sigma). The sections were examined using a BX41 epifluorescence microscope (Olympus, Poland), equipped with a filter set for visualization of Alexa Fluor 488 (excitation filter BP470-490, dichromatic mirror DM500, barrier filter BA520IF). Images were captured using an Olympus XC50 camera. To reveal the cellular pattern, immunolabeled sections were viewed and photographed using phase contrast optics. The intensity of labeling was evaluated according to Pielach et al. [84] and Potocka et al. [21] and expressed as no labeling (–), weak labeling (+), moderate labeling (++), strong labeling (+++) and very strong labeling (++++).

## 5. Conclusions

Suppression of protoplast recalcitrance would definitely bring a great progress in crop improvement. After the removal of the cell wall, the ability to re-synthesize it is the first and crucial event required to reactivate mitotic division and further development. To our knowledge, this was the first report presenting the spatio-temporal composition of the reconstituted cell wall in protoplast-derived cells of the *Daucus* and the role of phytosulfokine, a new peptidyl plant growth factor, in that process. We demonstrated that: (1) the differences in the chemical composition of the cell walls in the control and the PSK-treated cultures occurred, (2) the first chemical components of the wall reconstituted by protoplast-derived cells were pectins recognized by the LM20 antibody and the AGPs recognized by JIM8 and JIM13, (3) the distribution of pectins with different levels of methyl-esterification changed in a diverse manner with culture condition and species, (4) the expression and localization of AGPs in protoplast-derived cells and suspension aggregates were time and tissue specific and enhanced by the PSK, (5) the AGPs located in the periplasmic space might be involved in plasma membrane-cell wall interactions with signaling impacts, (6) an increase in the extensin level was characteristic for the middle stage of culture especially in PSK-treated cultures, (7) the used taxa showed a diverse response to PSK in terms of culture development and (8) PSK accelerated the process of wall re-synthesis by protoplast-derived cells. In our opinion, the analyzed epitopes might serve as markers of developmental potential of *Daucus* protoplast cultures with the following meaning: (1) the pectic (recognized by the LM19 and LM20 antibodies) and AGP epitopes (recognized by JIM4 and JIM13) are markers of new walls, and thus markers of emerging cell clusters, indicating the growth potential of the culture; (2) LM19 is a marker of PEM and the extracellular matrix covering the somatic embryos; (3) LM20 and JIM8 are markers of provascular tissue in somatic embryos; (4) the AGPs recognized by the JIM8 and JIM13 antibody are the markers of alive cells and (5) JIM4, JIM8, JIM13 and JIM12 are negative markers of PEM. We believed that the presented data added some new information about the role of chemical constitution of the cell wall in plant differentiation processes.

## Figures and Tables

**Table 1:** Isolation efficiency and viability of leaf-derived protoplasts within analyzed *Daucus* species.

Species	Protoplast Yield (× 10^6^/g FW)		Protoplast Viability (%)	
	Mean ± SE	*n*	Mean ± SE	*n*
*D. carota* subsp. *sativus* (cv. Dolanka)	5.1 ± 0.9 a	3	69.0 ± 2.3 a	3
*D. carota* subsp. *gadecaei*	5.2 ± 1.6 a	3	68.0 ± 1.3 a	3
*D. montevidensis*	1.3 ± 0.4 b	6	48.8 ± 2.7 b	4

FW fresh weight, *n* number of independent protoplast isolations; means within each column followed by different letters (a, b) are significantly different (*p* ≤ 0.05).

**Table 2:** Origin, seed source, somatic chromosome number (2n) and time of sampling for immunohistological analysis of three *Daucus* taxa used for protoplast cultures.

Species	Origin, Seed Source	2n	Time of Sampling for Epitope Analysis(day of pc)									
			0	1	2	3	4	5	10	20	30	60
*D. carota* subsp. *sativus*	Poland, OP, cv. Dolanka (Polan Spółka z o.o.)	18	**+**	**+**	**+**	**+**	**+**	**+**	**+**	**+**	**+**	**+**
*D. carota* subsp. *gadecaei*	UK, HRI 7160	18	**+**	**+**	**+**	**+**	**+**	**+**	**+**	**+**	**+**	**+**
*D. montevidensis*	South America, JKI AS162	22	**+**	**+**	**+**	**+**	**+**	**+**	**+**	**+**	**-**	**-**

OP = open pollinated cultivar; HRI = Horticulture Research Institute, Genetic Resources Unit (Wellesbourne, UK) numbers; JKI = Julius Kuhn Institute (Germany) numbers; pc = protoplast culture; + = samples collected at pointed day of culture; - = samples not collected (due to clear degeneration symptoms of the cells); the analyses were carried out at each time point indicated in the table; however, in the figures, representative images were shown only from those time points at which changes in the presence of the analyzed epitopes were observed in comparison to one day-old cultures (day 1).

**Table 3:** Primary antibodies used in the study.

Antibody	Epitope	References
Anti-pectin		
LM19	Low methyl-esterified HG	[58]
LM20	High metyl-esterifed HG	[58]
Anti-AGP		
JIM4	AGP glycan (betaGlcA-(1,3)-alphaGalA-(1,2)-Rha)	[55,85]
JIM8	Arabinogalactan (epitope structure unknown)	[86]
JIM13	AGP glycan ((beta)GlcA1->3(alpha)GalA1->2Rha)	[86]
Anti-extensin		
JIM12	(epitope structure unknown)	[27]

## Abbreviations

AGPs	Arabinogalactan proteins
CPP	Carrot petiole protoplast medium
HG	Homogalaturonan
HRGPs	Hydroxyproline-rich glycoproteins
mAbs	Monoclonal antibodies
MS	Murashige and Skoog medium (1962)
PEM	Proembryonic mass
PME	Pectic methyl-esterase activity
PSK	Phytosulfokine-α
TAL	Thin alginate layer
R	Regeneration medium

## References

[1] Plunkett G.M., Pimenov M.G., Reduron J.-P., Kljuykov E.V., van Wyk B.-E., Ostroumova T.A., Henwood M.J., Tilney P.M., Spalik K., Watson M.F., Kadereit J.W., Bittrich V. (2018). Apiaceae. Flowering Plants. Eudicots: Apiales, Gentianales (Except Rubiaceae).

[2] Spooner D.M., Simon P., Iorizzo M., Grzebelus D., Baranski R. (2019). Daucus: Taxonomy, Phylogeny, Distribution. The Carrot Genome. Compendium of Plant Genomes.

[3] Banasiak Ł., Wojewódzka A., Baczyński J., Reduron J.-P., Piwczyński M., Kurzyna-Młynik R., Gutaker R., Czarnocka-Cieciura A., Kosmala-Grzechnik S., Spalik K. (2016). Phylogeny of *Apiaceae* subtribe *Daucinae* and the taxonomic delineation of its genera. Taxon.

[4] Heywood V.H. (2014). The socio-economic importance of the Apiales. J. Fac. Pharm. Istambul.

[5] Gaurtheret R.J. (1939). Sur la possibilite de realiser La culture indefinite des tissues de tubercules de carotte. CR Hebd. Seances Acad. Sci..

[6] Steward F.C. (1958). Growth and organized development of cultured cells. III. Interpretations of the growth from free cell to carrot plant. Am. J. Bot..

[7] Eeckhaut T., Lakshmanan P.S., Deryckere D., Van Bockstaele E., Van Huylenbroeck J. (2013). Progress in plant protoplast research. Planta.

[8] Wang J., Jiang J., Wang Y. (2013). Protoplast fusion for crop improvement and breeding in China. Plant Cell Tissue Organ. Cult..

[9] Kameya T., Uchimiya H. (1972). Embryoids derived from isolated protoplasts of carrot. Planta.

[10] Grambow H.J., Kao K.N., Miller R.A., Gamborg O.L. (1972). Cell division and plant development from protoplasts of carrot cell suspension cultures. Planta.

[11] Arbizu C., Reitsma K.R., Simon P.W., Spooner D.M. (2014). Morphometrics of Daucus (*Apiaceae*): A counterpart to a phylogenomic study. Am. J. Bot..

[12] Dudits D., Hadlaczky G., Lévi E., Fejér O., Haydu Z., Lazar G. (1977). Somatic hybridisation of *Daucus carota* and *D. capillifolius* by protoplast fusion. Theor. Appl. Genet..

[13] Ichikawa H., Tanno-Suenaga L., Imamura J. (1987). Selection of *Daucus cybrids* based on metabolic complementation between X-irradiated *D. capillifolius* and iodoacetamide-treated *D. carota* by somatic cell fusion. Theor. Appl. Genet..

[14] Dirks R., Sidorov V., Tulmans C. (1996). A new protoplast culture system in *Daucus carota* L. and its applications for mutant selection and transformation. Theor. Appl. Genet..

[15] Grzebelus E., Szklarczyk M., Baranski R. (2012). An improved protocol for plant regeneration from leaf-and hypocotyl-derived protoplasts of carrot. Plant Cell Tissue Organ. Cult..

[16] Maćkowska K., Jarosz A., Grzebelus E. (2014). Plant regeneration from leaf-derived protoplasts within the *Daucus* genus: Effect of different conditions in alginate embedding and phytosulfokine application. Plant Cell Tissue Organ. Cult..

[17] Davey M.R., Anthony P., Power J.B., Lowe K.C. (2005). Plant protoplasts: Status and biotechnological perspectives. Biotechnol. Adv..

[18] Chakraborty S. (2016). Protoplast Culture and Somatic Hybridization, a Promising Frontier of Plant Biotechnology. J. Arts Sci. Teach..

[19] Majewska-Sawka A., Münster A. (2003). Cell-wall antigens in mesophyll cells and mesophyll-derived protoplasts of sugar beet: Possible implication in protoplast recalcitrance?. Plant Cell Rep..

[20] Sala K., Potocka I., Kurczynska E. (2013). Spatio-temporal distribution and methyl-esterification of pectic epitopes provide evidence of developmental regulation of pectins during somatic embryogenesis in *Arabidopsis thaliana*. Biol. Plant..

[21] Potocka I., Godel K., Dobrowolska I., Kurczyńska E.U. (2018). Spatio-temporal localization of selected pectic and arabinogalactan protein epitopes and the ultrastructural characteristics of explant cells that accompany the changes in the cell fate during somatic embryogenesis in *Arabidopsis thaliana*. Plant Physiol. Biochem..

[22] Malinowski R., Filipecki M. (2002). The role of cell wall in plant embryogenesis. Cell. Mol. Biol. Lett..

[23] David H., Savy C., Miannay N., Dargent R., David A. (1994). Supporting matrix influences protoplast-derived colony formation: Structural analysis. Protoplasma.

[24] Kwon H.-K., Yokoyama R., Nishitani K. (2005). A proteomic approach to apoplastic proteins involved in cell wall regeneration in protoplasts of Arabidopsis suspension-cultured cells. Plant Cell Physiol..

[25] Yang X., Tu L., Zhu L., Fu L., Min L., Zhang X. (2008). Expression profile analysis of genes involved in cell wall regeneration during protoplast culture in cotton by suppression subtractive hybridization and macroarray. J. Exp. Bot..

[26] Betekhtin A., Rojek M., Jaskowiak J., Milewska-Hendel A., Kwasniewska J., Kostyukova Y., Kurczynska E., Rumyantseva N., Hasterok R. (2017). Nuclear genome stability in long-term cultivated callus lines of *Fagopyrum tataricum* (L.) Gaertn. PLoS ONE.

[27] Betekhtin A., Rojek M., Nowak K., Pinski A., Milewska-Hendel A., Kurczynska E., Doonan J., Hasterok R. (2018). Cell wall epitopes and endoploidy as reporters of embryogenic potential in *Brachypodium distachyon callus* culture. Int. J. Mol. Sci..

[28] Butowt R., Niklas A., Rodriguez-Garcia M.I., Majewska-Sawka A. (1999). Involvement of JIM13-and JIM8-responsive carbohydrate epitopes in early stages of cell wall formation. J. Plant Res..

[29] Wiśniewska E., Majewska-Sawka A. (2008). The differences in cell wall composition in leaves and regenerating protoplasts of *Beta vulgaris* and *Nicotiana tabacum*. Biol. Plant..

[30] Matsubayashi Y., Takagi L., Sakagami Y. (1997). Phytosulfokine-α, a sulfated pentapeptide, stimulates the proliferation of rice cells by means of specific high-and low-affinity binding sites. Proc. Natl. Acad. Sci. USA.

[31] Grzebelus E., Szklarczyk M., Greń J., Śniegowska K., Jopek M., Kacińska I., Mrożek K. (2012). Phytosulfokine stimulates cell divisions in sugar beet (*Beta vulgaris* L.) mesophyll protoplast cultures. Plant Growth Regul..

[32] Davey M.R., An P., Power J.B., Lowe K.C. (2005). Plant protoplast technology: Current status. Acta Physiol. Plant..

[33] Davey M.R., Anthony P., Power J.B., Lowe K.C. (2005). 2004 SIVB congress symposium proceedings “Thinking outside the cell”: Plant protoplast technology: Status and applications. Vitr. Cell. Dev. Biol..

[34] Grzebelus E., Maćkowska K., Macko-Podgorni A., Kiełkowska A., Szklarczyk M., Baranski R., Grzebelus D. (2019). Application of protoplast technology to *Apiaceaae* species. Acta Hortic..

[35] Dudits D., Maroy E., Praznovszky T., Olah Z., Gyorgyey J., Cella R. (1987). Transfer of resistance traits from carrot into tobacco by asymmetric somatic hybridization: Regeneration of fertile plants. Proc. Natl. Acad. Sci. USA.

[36] Kisaka H., Kisaka M., Kanno A., Kameya T. (1997). Production and analysis of plants that are somatic hybrids of barley (*Hordeum vulgare* L.) and carrot (*Daucus carota* L.). Theor. Appl. Genet..

[37] Han L., Zhou C., Shi J., Zhi D., Xia G. (2009). Ginsenoside Rb 1 in asymmetric somatic hybrid calli of *Daucus carota* with *Panax quinquefolius*. Plant Cell Rep..

[38] Caffall K.H., Mohnen D. (2009). The structure, function, and biosynthesis of plant cell wall pectic polysaccharides. Carbohydr. Res..

[39] Liepman A.H., Wightman R., Geshi N., Turner S.R., Scheller H.V. (2010). *Arabidopsis*—A powerful model system for plant cell wall research. Plant J..

[40] Namasivayam P., Skepper J.N., Hanke D. (2010). Distribution of arabinogalactan protein (AGP) epitopes on the anther-derived embryoid cultures of *Brassica napus*. Pertanika J. Trop. Agric. Sci..

[41] Wiszniewska A., Piwowarczyk B. (2014). Studies on cell wall regeneration in protoplast culture of legumes—The effect of organic medium additives on cell wall components. Czech J. Genet. Plant Breed..

[42] Majewska-Sawka A., Münster A., Rodríguez-García M.I. (2002). Guard cell wall: Immunocytochemical detection of polysaccharide components. J. Exp. Bot..

[43] Verhertbruggen Y., Marcus S.E., Haeger A., Verhoef R., Schols H.A., McCleary B.V., McKee L., Gilbert H.J., Paul Knox J. (2009). Developmental complexity of arabinan polysaccharides and their processing in plant cell walls. Plant J..

[44] Wolf S., Mouille G., Pelloux J. (2009). Homogalacturonan methyl-esterification and plant development. Mol. Plant.

[45] Mock H., Emmerling M., Seitz H.U. (1990). Cell wall synthesis in carrot cells: Comparison of suspension-cultured cells and regenerating protoplasts. Physiol. Plant..

[46] Zhao J., Mollet J.-C., Lord E.M. (2004). Lily (*Lilium longiflorum* L.) pollen protoplast adhesion is increased in the presence of the peptide SCA. Sex. Plant Reprod..

[47] Moustacas A., Nari J., Diamantidis G., Noat G., Crasnier M., Borel M., Ricard J. (1986). Electrostatic effects and the dynamics of enzyme reactions at the surface of plant cells: 2. The role of pectin methyl esterase in the modulation of electrostatic effects in soybean cell walls. Eur. J. Biochem..

[48] David H., Bade P., David A., Savy C., Demazy C., Van Cutsem P. (1995). Pectins in walls of protoplast-derived cells imbedded in agarose and alginate beads. Protoplasma.

[49] Shea E.M., Gibeaut D.M., Carpita N.C. (1989). Structural analysis of the cell walls regenerated by carrot protoplasts. Planta.

[50] Hanai H., Matsuno T., Yamamoto M., Matsubayashi Y., Kobayashi T., Kamada H., Sakagami Y. (2000). A secreted peptide growth factor, phytosulfokine, acting as a stimulatory factor of carrot somatic embryo formation. Plant Cell Physiol..

[51] Rodakowska E., Kasprowicz A., Łapa A., Łuczak M., Derba M., Wojtaszek P. (2006). Ściany komórkowe jako źródło sygnałów regulujących procesy rozwojowe komórek roślin. Biotechnologia.

[52] Stührwohldt N., Dahlke R.I., Kutschmar A., Peng X., Sun M., Sauter M. (2015). Phytosulfokine peptide signaling controls pollen tube growth and funicular pollen tube guidance in *Arabidopsis thaliana*. Physiol. Plant..

[53] Hartmann J., Stührwohldt N., Dahlke R.I., Sauter M. (2013). Phytosulfokine control of growth occurs in the epidermis, is likely to be non-cell autonomous and is dependent on brassinosteroids. Plant J..

[54] Pennell R.I., Janniche L., Kjellbom P., Scofield G.N., Peart J.M., Roberts K. (1991). Developmental regulation of a plasma membrane arabinogalactan protein epitope in oilseed rape flowers. Plant Cell Online.

[55] Knox J.P., Day S., Roberts K. (1989). A set of cell surface glycoproteins forms an early position, but not cell type, in the root apical carota L.. Development.

[56] Knox J.P., Linstead P.J., Cooper J.P., Roberts K. (1991). Developmentally regulated epitopes of cell surface arabinogalactan proteins and their relation to root tissue pattern formation. Plant J..

[57] Schindler T., Bergfeld R., Schopfer P. (1995). Arabinogalactan proteins in maize coleoptiles: Developmental relationship to cell death during xylem differentiation but not to extension growth. Plant J..

[58] Sala K., Malarz K., Barlow P.W., Kurczyńska E.U. (2017). Distribution of some pectic and arabinogalactan protein epitopes during *Solanum lycopersicum* (L.) adventitious root development. BMC Plant Biol..

[59] Stacey N.J., Roberts K., Knox J.P. (1990). Patterns of expression of the JIM4 arabinogalactan-protein epitope in cell cultures and during somatic embryogenesis in *Daucus carota* L.. Planta.

[60] Šamaj J., Baluška F., Bobák M., Volkmann D. (1999). Extracellular matrix surface network of embryogenic units of friable maize callus contains arabinogalactan-proteins recognized by monoclonal antibody JIM4. Plant Cell Rep..

[61] Serpe M.D., Nothnagel E.A. (1994). Effects of Yariv phenylglycosides on *Rosa* cell suspensions: Evidence for the involvement of arabinogalactan-proteins in cell proliferation. Planta.

[62] Willats W.G.T., Knox J.P. (1996). A role for arabinogalactan-proteins in plant cell expansion: Evidence from studies on the interaction of β-glucosyl Yariv reagent with seedlings of *Arabidopsis thaliana*. Plant J..

[63] Majewska-Sawka A., Nothnagel E.A. (2000). The multiple roles of arabinogalactan proteins in plant development. Plant Physiol..

[64] Wiśniewska E., Majewska-Sawka A. (2007). Arabinogalactan-proteins stimulate the organogenesis of guard cell protoplasts-derived callus in sugar beet. Plant Cell Rep..

[65] Kreuger M., van Holst G.-J. (1993). Arabinogalactan proteins are essential in somatic embryogenesis of *Daucus carota* L.. Planta.

[66] Jauh G.Y., Lord E.M. (1996). Localization of pectins and arabinogalactan-proteins in lily (*Lilium longiflorum* L.) pollen tube and style, and their possible roles in pollination. Planta.

[67] Pereira A.M., Pereira L.G., Coimbra S. (2015). Arabinogalactan proteins: Rising attention from plant biologists. Plant Reprod..

[68] Pennell R.I., Roberts K. (1990). Sexual development in the pea is presaged by altered expression of arabinogalactan protein. Nature.

[69] Betekhtin A., Rojek M., Milewska-Hendel A., Gawecki R., Karcz J., Kurczynska E., Hasterok R. (2016). Spatial distribution of selected chemical cell wall components in the embryogenic callus of *Brachypodium distachyon*. PLoS ONE.

[70] Shibaya T., Sugawara Y. (2009). Induction of multinucleation by β-glucosyl Yariv reagent in regenerated cells from *Marchantia polymorpha* protoplasts and involvement of arabinogalactan proteins in cell plate formation. Planta.

[71] Lamport D.T.A., Kieliszewski M.J., Chen Y., Cannon M.C. (2011). Role of the extensin superfamily in primary cell wall architecture. Plant Physiol..

[72] Smallwood M., Beven A., Donovan N., Neill S.J., Peart J., Roberts K., Knox J.P. (1994). Localization of cell wall proteins in relation to the developmental anatomy of the carrot root apex. Plant J..

[73] Parmentier Y., Durr A., Marbach J., Hirsinger C., Criqui M.-C., Fleck J., Jamet E. (1995). A novel wound-inducible extensin gene is expressed early in newly isolated protoplasts of *Nicotiana sylvestris*. Plant Mol. Biol..

[74] Casero P.J., Casimiro I., Knox J.P. (1998). Occurrence of cell surface arabinogalactan-protein and extensin epitopes in relation to pericycle and vascular tissue development in the root apex of four species. Planta.

[75] Cosgrove D.J. (2005). Growth of the plant cell wall. Nat. Rev. Mol. Cell Bio..

[76] Yu L., Liu Y., Liu Y., Li Q., Tang G. (2016). Overexpression of phytosulfokine-α induces male sterility and cell growth by regulating cell wall development in *Arabidopsis*. Plant Cell Rep..

[77] Wolf S., van der Does D., Ladwig F., Sticht C., Kolbeck A., Schürholz A.K., Augustin S., Keinath N., Rausch T., Greiner S. (2014). A receptor-like protein mediates the response to pectin modification by activating brassinosteroid signaling. Proc. Natl. Acad. Sci. USA.

[78] Holzwart E., Huerta A.I., Glöckner N., Gómeza B.G., Wanke F., Augustin S., Askani J.C., Schürholz A.K., Harter K., Wolf S. (2018). BRI1 controls vascular cell fate in the *Arabidopsis* root through RLP44 and phytosulfokine signaling. Proc. Natl. Acad. Sci. USA.

[79] Schürholz A.K. (2019). Spatio-temporal control of cell wall properties and signalling networks in *Arabidopsis* meristems. Ph.D. Thesis.

[80] Gómez B.G. (2017). Phosphorylation of RLP44: Shifting between subcellular localization and receptor complexes. Ph.D. Thesis.

[81] Gancheva M.S., Malovichkoa Y.V., Poliushkevich L.O., Dodueva I.E., Lutova L.A. (2019). Plant peptide hormones. Russ. J. Plant Physiol..

[82] Murashige T., Skoog F. (1962). A revised medium for rapid growth and bio assays with tobacco tissue cultures. Physiol. Plant..

[83] Anthony P., Davey M.R., Power J.B., Lowe K.C. (1997). Enhanced mitotic division of cultured Passiflora and Petuniaprotoplastsby oxygenated perfluorocarbon and haemoglobin. Biotechnol. Tech..

[84] Pielach A., Leroux O., Domozych D.S., Knox J.P., Popper Z.A. (2014). Arabinogalactan protein-rich cell walls, paramural deposits and ergastic globules define the hyaline bodies of rhinanthoid *Orobanchaceae haustoria*. Ann. Bot..

[85] Yates E.A., Valdor J.-F., Haslam S.M., Morris H.R., Dell A., Mackie W., Knox J.P. (1996). Characterization of carbohydrate structural features recognized by anti-arabinogalactan-protein monoclonal antibodies. Glycobiology.

[86] Milewska-Hendel A., Baczewska A.H., Sala K., Dmuchowski W., Brągoszewska P., Gozdowski D., Jozwiak A., Chojnacki T., Swiezewska E., Kurczynska E. (2017). Quantitative and qualitative characteristics of cell wall components and prenyl lipids in the leaves of *Tilia* x *euchlora* trees growing under salt stress. PLoS ONE.

